# Shopper intent prediction from clickstream e-commerce data with minimal browsing information

**DOI:** 10.1038/s41598-020-73622-y

**Published:** 2020-10-12

**Authors:** Borja Requena, Giovanni Cassani, Jacopo Tagliabue, Ciro Greco, Lucas Lacasa

**Affiliations:** 1 ICFO - Institut de Ciencies Fotoniques, The Barcelona Institute of Science and Technology, Av. Carl Friedrich Gauss 3, 08860 Castelldefels, Barcelona Spain; 2 Department of Cognitive Science and Artificial Intelligence, Tilburg University, Warandelaan 2, 5037 AB Tilburg, The Netherlands; 3 Coveo Labs, 44 Montgomery Street, San Francisco, CA 94105 USA; 4 School of Mathematical Sciences, Queen Mary University of London, Mile End Road, London, E14NS UK

**Keywords:** Computational science, Computer science

## Abstract

We address the problem of user intent prediction from clickstream data of an e-commerce website via two conceptually different approaches: a hand-crafted feature-based classification and a deep learning-based classification. In both approaches, we deliberately coarse-grain a new clickstream proprietary dataset to produce symbolic trajectories with minimal information. Then, we tackle the problem of trajectory classification of arbitrary length and ultimately, early prediction of limited-length trajectories, both for balanced and unbalanced datasets. Our analysis shows that *k*-gram statistics with visibility graph motifs produce fast and accurate classifications, highlighting that purchase prediction is reliable even for extremely short observation windows. In the deep learning case, we benchmarked previous state-of-the-art (SOTA) models on the new dataset, and improved classification accuracy over SOTA performances with our proposed LSTM architecture. We conclude with an in-depth error analysis and a careful evaluation of the pros and cons of the two approaches when applied to realistic industry use cases.

## Introduction

Alice lands on *myFashionShop.com* and starts browsing for running shoes: is she going to buy something in the end, or will she leave without purchasing anything? Intuitively, the more she interacts with the website, the more data we collect, the easier it should be to answer that question – but how easy *exactly*? Predicting whether Alice’s browsing session on *myFashionShop.com* will eventually end in a purchase is known as the *clickstream prediction* challenge^1–5^. The challenge is relevant to a broad audience, both from a theoretical and practical perspectives. On the theoretical side, it is a particular instance of the fundamental problem of feature-based classification of sequences^6^. Compared to other use cases, such as protein sequences^7^ or EEG^8^ signal processing, the scale of web inferences, dataset properties and time constraints make clickstream prediction stand out as a particularly challenging scenario to test old and new statistical tools. On the practical side, the growth of online retail^9^—with approximately 25% of all fashion-related transactions now happening on digital shops^10^—and the connection between browsing behavior and an entire ecosystem of applied science problems^11^, makes intent prediction a very valuable problem to solve.

In this work we extensively study the clickstream prediction problem leveraging a new dataset, which contains rich clickstream data on online users browsing a popular fashion e-commerce website: given a set of interactions in an e-commerce shop, can we predict whether the sequence will eventually contain a purchase event? Our systematic investigation on intent prediction serves as both a baseline for future iterations and as an example of the variety of analyses offered by the data source, which we plan to release to the scientific community with a research-friendly license as part of this publication. With this goal in mind, we tackle two specific tasks—(1) the classification of arbitrarily long click sequences and (2) the early prediction of limited-length sequences—by comparing two radically different strategies involving two alternative algorithmic approaches: (1) using classification algorithms on a set of pre-defined hand-crafted features, and (2) learning the features via deep learning (DL). The former is computationally efficient, scalable and has interpretable features, while the second is more sophisticated and involves non-interpretable features. In (1) we make use of classical symbolic features, such as *k*-grams, and complement them with combinatorial features recently introduced in the complex systems community, such as horizontal visibility graph motifs (HVGm)^12^. To the best of our knowledge, we detail the first end-to-end HVGm-based pipeline to analyse online user behavior. As for (2), we extend and improve state of the art deep learning models^4^, by extensively benchmarking alternative Long Short-Term Memory (LSTM) architectures.

Overall, our results suggest that prediction of user intent is already possible with minimal information on their navigation patterns. Besides, early classification is also possible even for very short sequences. Our quantitative comparison highlights precisely the trade-off between the two algorithmic approaches, so that industry decisions can be made with known bounds on performance. In particular, establishing new state-of-the-art results on this task^4^ can be achieved through deep neural nets at the expense of longer training time and a more costly infrastructure. Since no size fits all, and different players have different constraints, we believe that the thorough examinations in this contribution make for a valuable guide for practical engineering work in a variety of prediction scenarios.

Interestingly enough, we also note that previous research on the clickstream challenge suffers from low external validity^13^, as experiments on online behavior have been almost exclusively made on datasets that do not represent the vast majority of digital shops. Our observations, together with the release of data to the research community, take place in times of general concern regarding reproducible and ecologically valid^14^ research in the broad field of machine learning, as a significant portion of research is done by few private institutions leveraging data and computational power not available to the vast majority of society^15^. By releasing data that is more representative of typical players in online industries, we hope to foster a more inclusive and fruitful research agenda on the clickstream prediction and related behavioral challenges.

## Preliminaries

**Figure 1:**
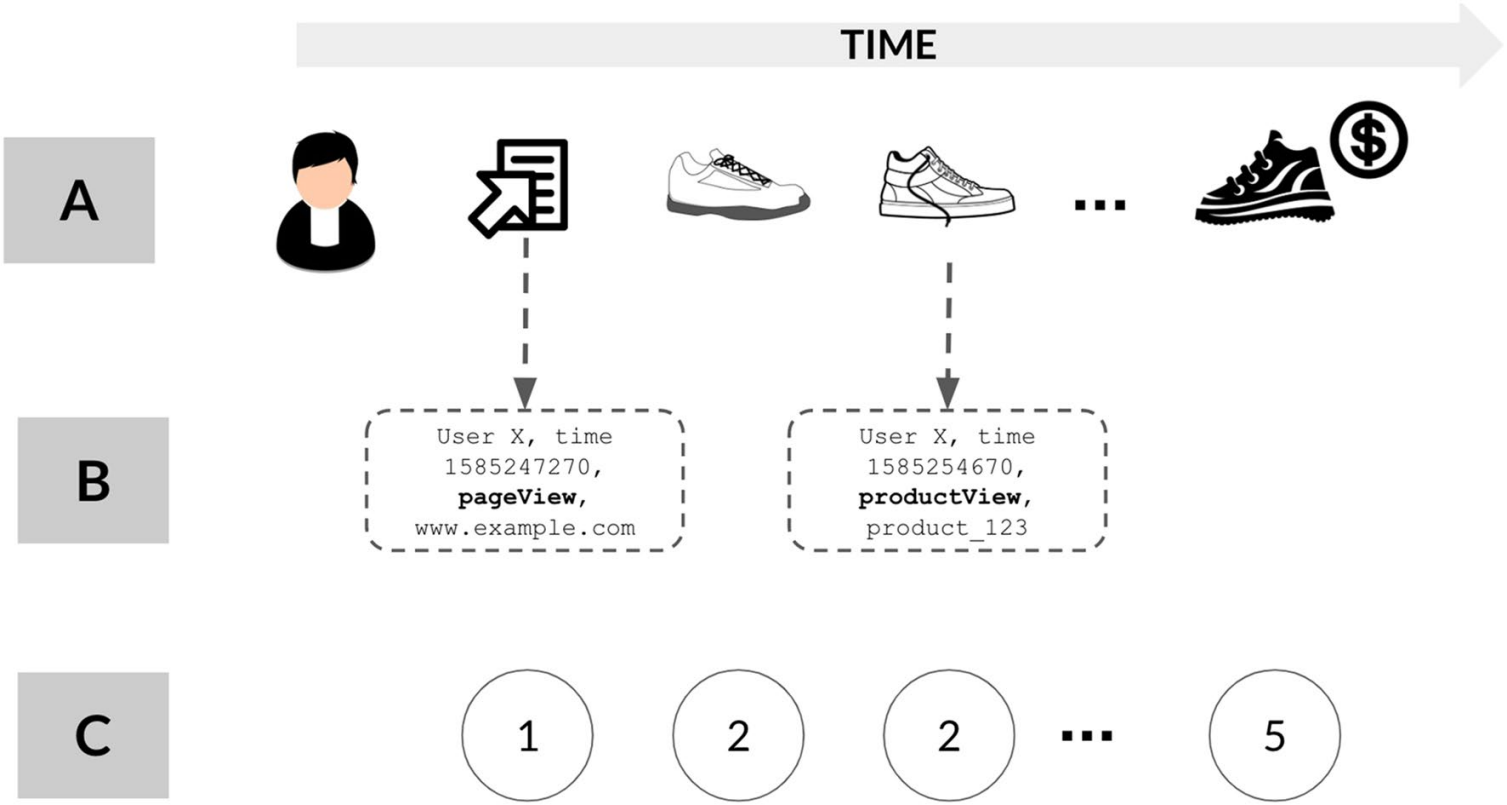
Alice sample session and high-level view of the clickstream prediction challenge. We can distinguish three layers of representation: **(A)** is the actual session, and we are asked to predict that the session ends with a purchase based on the browsing pattern; **(B)** is the rich meta-data layer, which comprises, for each interaction between Alice and the website, information about time, website location, product properties; **(C)** is a symbolized, minimal information layer, in which all meta-data about the products is stripped and only event types are retained (see section "Data") for details on the final dataset). We target only **(C)** in our experiments, therefore providing a strong benchmark for similar analyses focusing at level **(B)**.

Consider Alice’s session on *myFashionShop.com* as depicted in Fig. 1A. As the shopper is browsing, data about interactions with the website is collected in real-time and can be used to make the clickstream prediction: is Alice going to buy something before the end of her session? While the dataset we present is rich (Fig. 1B, see also section "Data"), the focus of *this* investigation is the “symbolized” version of Alice’s session (Fig. 1C), that is, a sequence containing minimal information. A shopper browsing an e-commerce website is similar to a walker navigating a network enriched with metadata: the shopper generates a *trajectory* hopping between nodes, with a certain amount of time between hops. Focusing on symbolized trajectories implies discarding substantial information on their associated metadata. Hence, user intent prediction boils down to whether these minimal trajectories enclose stylized patterns significant enough to solve the classification problem.

Since the aim of this work is not just presenting state-of-the-art performance on a target dataset, but also placing the clickstream prediction task into a broader context, we believe that presenting extensive analysis on *symbolized* sequences is valuable for three reasons. The first is parsimony, as we wish to disentangle the information provided by the repetition of clickstream patterns from other relevant information, such as time duration between clicks, network topological properties of the nodes visited along the trajectories, etc. Second, removing the aforementioned metadata could make the prediction problem *more* challenging, so that that our results can be used as a benchmark for further analyses incorporating additional information from the original dataset. Third, validating low complexity and flexible setups is a key requirement in the quest for real-time implementations and in online marketing’s decision-making processes. Such approaches come with an additional trait: they are less platform-dependent and are, therefore, easier to implement for a wider range of applications in which fine-grained data tracking may not be available or reliable.

A second important point about the underlying motivation and philosophy of the proposed work is related to the external validity of sequence prediction algorithms for digital online behavior, which appear to pose different challenges to researchers than what is typically encountered in other superficially similar tasks—e.g. classification of ECG^16^ signals from patients with certain diseases or the characterization of complex physical phenomena^17^. In particular, purchase prediction in online shops is linked to an important business metric known as *conversion rate*, that is, the ratio between the number of sessions in which an item is purchased *vs.* the total number of sessions, within a given time window. Conversion rate is known to change drastically between countries, industries and even players^18^. As a relevant example, the state-of-the-art model proposed in^4^ was developed and benchmarked on one of the largest digital shops in the world, boasting a conversion rate of more than 20% (i.e., one out of five sessions actually contains a purchase event). On the contrary, the conversion rate on the dataset of this work (after cleaning and pre-processing) is at less than 5%. This disparity not only makes class imbalance worse, but also challenges the widespread validity of results obtained on very unusual datasets. Faithful to the overall effort in charting precisely the prediction landscape for this task, and not just providing “algorithmic recipes”, we conduct two *types* of analyses to deepen our understanding of the problem space.

Our first research question sits at the “computational” level (to borrow the famous distinction from^15^): in such a tiny state-space of symbolized trajectories, how much information is there *at all* and how are different strategies able to exploit it (e.g., discriminative *vs*. generative models)? To answer this question, we level the playing field and decouple any question about the conversion rate of particular shops from the question about symbolic prediction. In this vein, our results on a balanced version of the dataset provide an intuitive understanding of how challenging is the task at an abstract level, and give us insights on the robustness of several approaches that could be easily compared with literature from other sequence-based tasks.

Our second research question sits closer to the actual *implementation* level: how likely are the proposed methods to work in practice in industry scenarios which have very different data constraints? Based on ample experience in the e-commerce domain, we provide extensive benchmarks obtained by *systematically* moving away from a balanced dataset and towards a heavily imbalanced one. We believe this systematic approach has two important advantages over many research in machine learning as applied to e-commerce use cases. First, it removes dataset biases, such as conversion events in^4^, and allows us to benchmark methods proposed by several researchers in a consistent and unified framework. Second, it substitutes a “point estimate” on a specific dataset with a more nuanced understanding of the performance of the proposed methods under several conditions, providing practitioners working under different constraints with a better understanding of the task *in context*.

While the particular methods here proposed can surely be improved in future research, we hope *this* contribution serves also as a valuable map to guide academic and industry practitioners when doing systematic research in traditional and neural methods for sequence classification. By addressing both the “computational” and “implementation” question, our extensive benchmarks with minimal sequence information provide solid foundations for further work on the clickstream prediction challenge, as well as helping promoting good practices in reproducible research for online systems.

## Data

### Raw database

**Figure 2:**
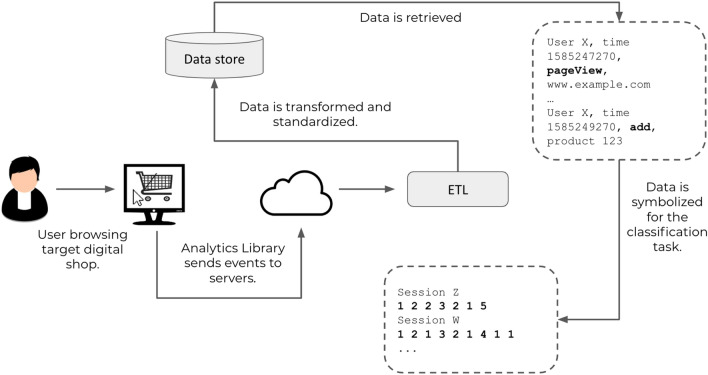
High-level workflow used in this work: a target dataset was created from real-time user data. Each event is subsequently mapped into a list of identifiers (symbolization process, see Table 1) and the resulting sequences are then used for machine learning analyses.

Raw data is kindly provided for this analysis by Coveo, a North American company providing A.I. services to the retail and service industries. As part of its services, the company collects data on partnering digital properties—typically e-commerce websites—through its own open source analytics library, in compliance with existing legislation^19^. The client library sends anonymized information about events on target properties such as users browsing pages, adding items to cart, buying them and so on to Coveo servers in real time. Figure 2 shows the high-level flow of information, from end users browsing on a partnering website, to the final dataset after metadata and user information have been removed.

The raw dataset for this study consists of browsing data retrieved from a popular fashion e-commerce website over the course of a two-month period in 2018. The data is stored and then anonymized by removing any individual identifier and accessory metadata, so that it is not possible to match events to neither specific products nor individual users. It should be noted that, for this study, users get assigned random identifiers, so that it is not even possible to re-identify a user as the same across different sessions.

Finally, in order to extract the trajectories, all events within the target period underwent a “sessionization” phase in which each one is assigned to a “session”, that is, a series of events that all happen within a certain time threshold. For this study, the industry standard of 30 minutes is chosen as the session threshold, i.e., if two events, A and B, produced by the same user are more than 30 minutes apart, a new session is created. After sessionization, trajectories are sampled at random from the resulting dataset in order to protect the data provider from revealing the total amount of traffic on the target website. As a result, the dataset is composed of 443, 652 anonymized clickstream trajectories of real customers. The next pre-processing step, symbolization, strips all but the most basic event information.

### Trajectory symbolization, definition of classes and trimming

In order to minimize the amount of information accompanying each trajectory, we apply a symbolization procedure in which each action triggered by the user is assigned a value according to the rule depicted in Table 1. For readers unfamiliar with the e-commerce scenario, we briefly survey event types: a *page view* is triggered when the final user loads any page in the website; a *detail* event is triggered when the user visits a specific product page, i.e. a page displaying one particular product; an *add/remove* event is triggered when a product is added/removed from the cart; a *purchase* event is triggered when the user actually buys the products in the cart; finally, a *click* event is triggered when some results from a search query are clicked, e.g. user searches for “shoes” using the e-commerce search bar, several products are returned and the user clicks on the second product triggering a *click* event.

**Table 1:** Symbol assignment rule by which a rich metadata trajectory is mapped into a simple symbolic sequence.

Action	Symbol
Page view	1
Detail (user sees product page)	2
Add (add product to cart)	3
Remove (remove product from cart)	4
Purchase (buy a product)	5
Click (click on result *after* search has been performed)	6

Hence, the problem of purchase prediction in a session of *n* actions translates into predicting the appearance of symbol 5 in the symbolized trajectories ${{\mathcal {S}}}(n)=(s_1,s_2,\dots ,s_n)$, where $s_i\in \{1,2,\dots ,6\}$. Each trajectory, therefore, is assigned to one of two possible classes: the *conversion* class (C) and the *non-conversion* class (NC). The conversion class C is assigned to all the trajectories which, at a given point, have a Purchase event, that is, trajectories that incorporate the symbol 5 at least once. NC, on the other hand, encompasses all the other trajectories, which means that they are generated by customers that navigate the website for some time and abandon the session without purchasing anything. In the raw dataset, out of the 443652 trajectories, 9212 ($2.08\%$) belong to class C and 434440 ($97.92\%$) belong to the class NC.

**Figure 3:**
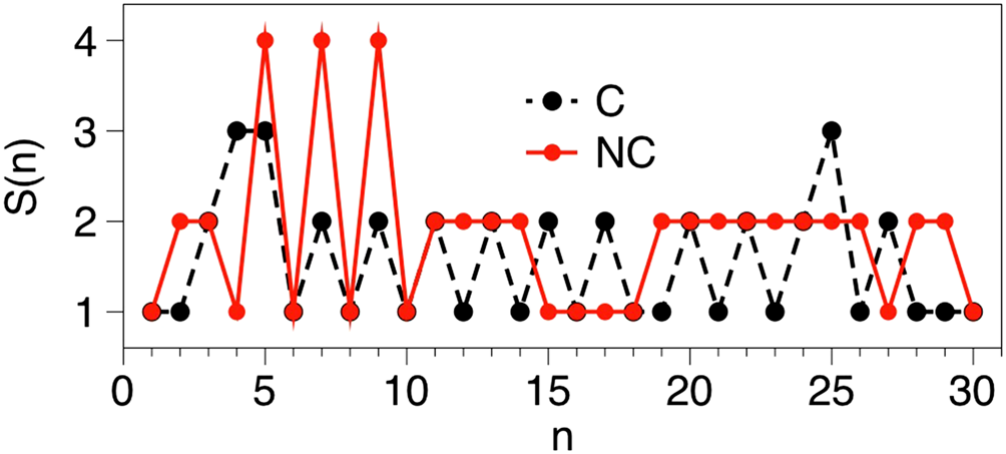
Sample of the first 30 points of clickstream symbolic trajectories ${{\mathcal {S}}}(n)$, one associated to a customer who won’t end up purchasing (NC, in red) and one associated to a customer who will eventually purchase (C, in black). Note that the Y axis is symbolic—each number corresponds to a different action, according to Table 1.

Now, in order to remove the class information from the observation, all the class C trajectories are trimmed—only the initial part of the trajectory that precedes the *first* appearance of a Purchase event remains. As a result, all the trajectories in our pre-processed dataset lack such event, yet all of them are tagged with their corresponding label (C or NC). Examples of a typical trajectory of each class are depicted in Fig. 3. Note that different symbolization rules, or just a permutation of the one depicted in Table 1, would yield different projected trajectories: in this work we follow the rules depicted in Table 1 but we recall that other assignments are equally possible.

### Task-specific datasets

As previously mentioned, we tackle two main prediction tasks: the classification of whole trajectories as they are, and the early prediction of such trajectories using only the information provided by the first *T* clicks. In order to do so, we generate different sub-datasets to perform the necessary conditioning of the clickstreams for each of the tasks.

#### Dataset (A)

For the whole trajectory classification, we only keep trajectories with length $L\ge 5$ clicks. This way, we ensure that trajectories are long enough to provide statistically meaningful information in the extracted features. The resulting dataset contains 203080 trajectories: 8324 ($4.10\%$) belonging to class C and 194756 ($95.90\%$) belonging to class NC.

#### Dataset (B)

For the early classification experiment (section "Early prediction"), we, again, consider only trajectories with length $L\ge 5$ clicks. Then, for each early window *T* to be considered, we generate a new sub-dataset taking the trajectories with length $L\ge T$ and trimming them all to length $L=T$.

In both cases, we also removed from these datasets the trajectories that are excessively long to be considered generated by humans given the 30 min session length. Thus, we take trajectories with length $L\le 155$ clicks, which implies a $1\%$ drop of Dataset (A).

## Hand-crafted, feature-based classification

In this section we describe the different descriptors that we extract from the trajectories in the context of feature engineering and we present the results of feature-based classification.

### *k*-grams

The most straightforward feature that one can extract from a symbolized time series is the joint distribution of symbols. A *k*-gram^20^ is defined as a block of *k* consecutive symbols. As such, isolated symbols are 1-g, whereas blocks of two consecutive symbols such as ‘11’, ‘12’, ‘13’, ..., ‘66’ are examples of 2-g. Notice that the symbol ‘5’ associated to the Purchase action will not appear in both 1- and 2-g by construction.

The (normalised) frequency histogram of 1-g is labelled *P*(*s*), whereas in general the (normalized) frequency histogram of *k*-grams is a joint probability distribution $P(s_1,s_2,\dots , s_k)$. Asymptotically, the set of *k*-grams distributions $\{P(s), P(s_1,s_2),\dots , P(s_1,s_2,\dots , s_k)\}$ would provide all the information of the hypothetical dynamical process generating the trajectories. As a matter of fact, the so-called Shannon’s entropy rate of such a process is defined as the limit

$$\begin{aligned} H=\lim _{k\rightarrow \infty }\frac{-1}{k}\sum _{{\mathbb {B}}(k)} P(s_1,\dots , s_k) \log P(s_1, \dots , s_k), \end{aligned}$$

where ${\mathbb {B}}(k)$ enumerates all blocks of size *k* (i.e. all possible *k*-grams). The entropy rate may be used to estimate the complexity of the process underpinning the observed trajectories. However, it is important to note that this quantity is, in practice, hard to compute, as the number of possible *k*-grams increases exponentially. For instance, in our case, where we have five symbols (1, 2, 3, 4 and 6), there is a total of $5^k$ possible different *k*-grams. Therefore one would need extremely long trajectories to accurately estimate even low order approximations to *H*. For these reasons, given typical session size in e-commerce applications, one can only look at short *k*-grams, usually for $k=1,2$.

**Figure 4:**
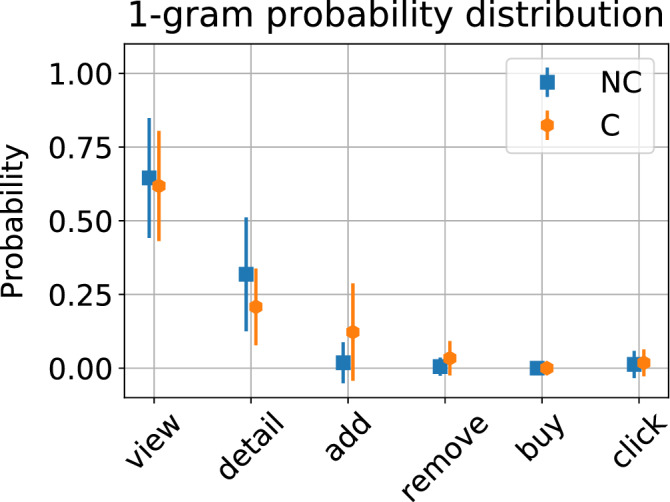
Mean normalized frequencies of 1 g, averaged over each class ensemble for trajectories of length $5\le L \le 155$. Error bars denote ± one standard deviation. This result shows that the relative abundance of each symbol is not a priori a fully discriminative feature of each class and, therefore, higher order statistics are required.

For parsimony, the first question to solve is whether the relative abundance of isolated symbols (1-g) is already a good discriminative feature of the two classes, as in that case there is no need to look at more sophisticated patterns in the data. A quick inspection of the trajectory samples depicted in Fig. 3 might suggest several possibilities: (1) the symbol ‘1’ (view) appears equally often in both classes, (2) the symbol ‘2’ (detail) seems to be overrepresented in the NC class, (3) the symbol ‘3’ (add) seems to be absent in the NC class, (4) there are no ‘6’ symbols (click) in either of the two samples. All of these possibilities are easily evaluated by looking at the average abundance of each symbol in each class, as shown in Fig. 4, where we plot the estimated *P*(*s*) calculated for each trajectory and averaged over classes. These preliminary results suggest that, while some subtle differences seem to be present for view and detail events, within error bars both classes have the same abundance of each; thus, 1-g statistics are not discriminative.

**Figure 5:**
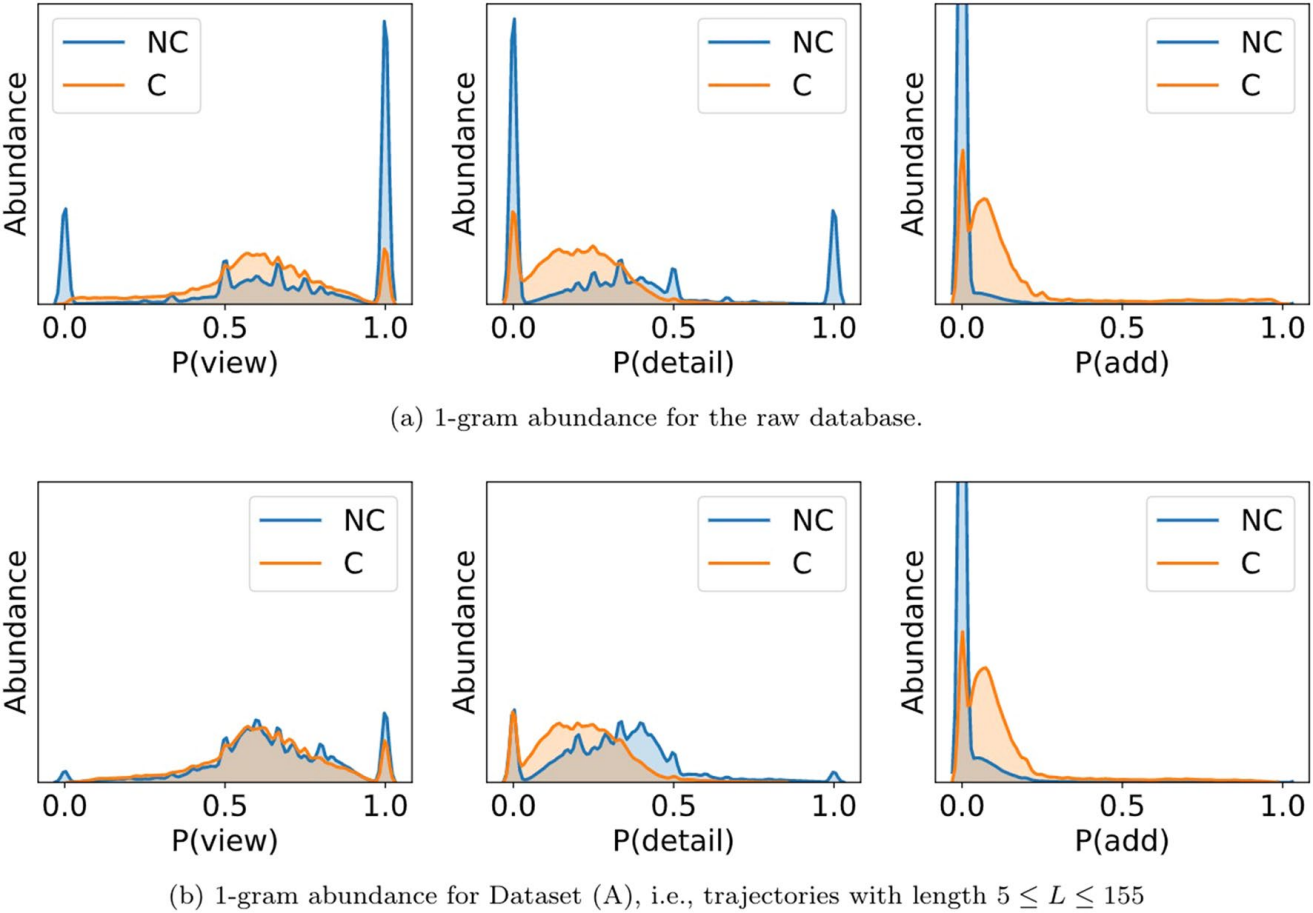
Intraclass distribution of frequencies for three 1-g: $P(\text {view})=P(1)$ (left panel), $P(\text {detail})=P(2)$ (middle panel) and $P(\text {add})=P(3)$ (right panel). In every case, we find that the symbols show up with comparable distributions in both classes. Top panels are based on the raw dataset, whereas the bottom panels are based on Dataset (A). We can see that the main difference between top and bottom panels relates to the fact that view and detail events are often overrepresented in very short sequences.

In order to explore this aspect in further depth, in Fig. 5 we plot the ensemble distributions of $P(\text {view})=P(1)$ (left panel), $P(\text {detail})=P(2)$ (middle panel) and $P(\text {add})=P(3)$ (right panel). Each panel depicts the distribution of how frequently a symbol shows up in each class, meaning that each curve displays the number of trajectories within a particular class displaying a certain *P*(*s*). Note that the upper panels are constructed using the raw data, whereas the bottom panels use only trajectories in Dataset (A) (section "Task-specific datasets"). This finer analysis confirms that the isolated actions view and detail appear with comparable frequencies in the two classes and are overrepresented in very short trajectories. On the other hand, the add action is more abundant in trajectories belonging to the C class, meaning that $P(3)=P(\text {add})$ is a potentially informative feature. It makes sense, provided that adding a product to the cart is a prerequisite for buying.

Accordingly, we extract 1-g and 2-g distributions *P*(*s*) and $P(s_1,s_2)$ as possible predictors of conversion. Now, it is clear from Fig. 3 that higher order statistics are needed. Since the nature of the data (relatively short trajectories) precludes extracting meaningful estimations of higher order *k*-grams, in the next section we introduce a simple and computationally efficient combinatorial metric which is devised to extract higher order patterns from short samples: horizontal visibility graph motifs (HVGm).

### Horizontal visibility graph motifs

**Figure 6:**
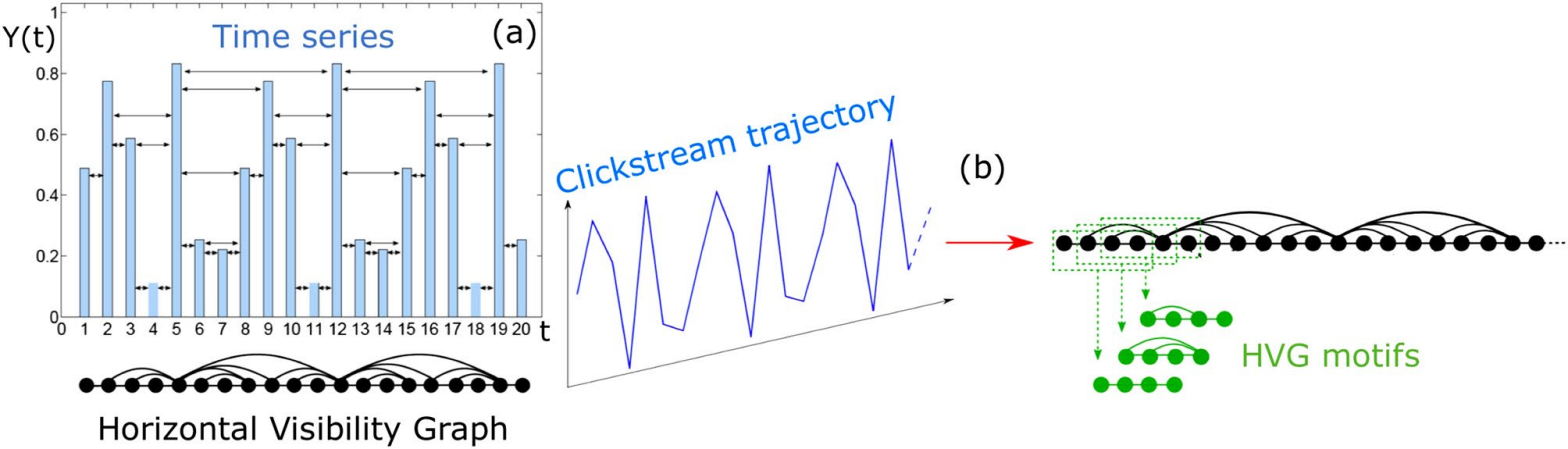
Panel (**a**) described how a time series *Y*(*t*) is converted into a Horizontal Visibility Graph (HVG) according to the visibility criterion. Panel (**b**) schematizes the visibility graph motif detection: a certain clickstream trajectory is mapped into an HVG and, from this, a window of size $n=4$ slides along the Hamiltonian path of the graph extracting the different HVG motifs.

A time series of *N* points can be transformed into a so-called *horizontal visibility graph* (HVG) of *N* nodes via the so-called visibility algorithm^21,22^. This is a method that enables the characterisation of time series and their underlying dynamics using combinatorics and graph theory. Additionally, the family of *sequential horizontal visibility graph motifs* (HVGm) has been recently introduced^12,23^. This latter method assesses the abundance of small combinatorial structures within a given time series. Below we provide definitions of HVG and HVGm (see Fig. 6 for an illustration):

#### Definition

*(HVG).* Let ${{\mathcal {S}}}=\{x_1,\dots ,x_N\}$, $x_i \in {\mathbb {R}}$ be a real-valued scalar time (or otherwise ordered) sequence of *N* data. Its horizontal visibility graph HVG(${{\mathcal {S}}}$) is defined as an undirected graph of *N* vertices, where each vertex $i\in \{1,2,\dots ,N\}$ is labelled in correspondence with the ordered datum $x_i$. Hence $x_1$ is related to vertex $i=1$, $x_2$ to vertex $i=2$, and so on. Then, two vertices *i*, *j* (assume $i<j$ without loss of generality) share an edge if and only if $x_k<\min (x_i,x_j),\ \forall k: i<k<j$.

HVG implements an ordering criterion which can be visualized in Fig. 6 (see^21^ for a convexity criterion that generates ‘natural’ visibility graphs instead). As noted above, visibility graphs and horizontal visibility graphs were introduced with the aims of using the tools of Graph Theory and Network Science^24^ to describe the structure of time series and their underlying dynamics from a combinatorial perspective (other proposals for graph-theoretical time series analysis can be found in^25,26^ and references therein and the extension of visibility graphs to image processing can be found in^27^). Research on this methodology has been primarily theoretical, elaborating on mathematical methods^28–31^ to extract rigorous results on the properties of these graphs when associated to canonical models of complex dynamics, including stochastic processes with and without correlations or chaotic processes^32–35^. In practice, this method can be used as a feature extraction procedure for constructing feature vectors for statistical learning purposes and has been widely applied across the disciplines (see, for instance,^36–40^ for just a few examples).

Taking into account that the prototypical trajectory analysed in this work is a short one, it is more likely that local patterns might provide more information than global metrics. In the context of HVGs, in order to examine the local abundance of small subgraphs within an HVG the concept of sequential HVG motifs was recently introduced^12^, namely:

#### Definition

(*HVGm of order p*) Consider a HVG of *N* nodes, associated to a time series of *N* data, and label the nodes according to the natural ordering induced by the arrow of time (i.e. the trivial Hamiltonian path). Set $p<N$ and consider, sequentially, all the subgraphs formed by the sequence of nodes $\{i,i+1,\dots ,i+p-1\}$ (where *i* is an integer that takes values in $\{1,N-p+1\}$) and the edges from the HVG only connecting these nodes: these are defined as the sequential HVG motifs of order *p*.

**Figure 7:**
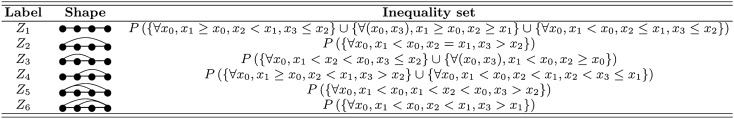
Enumeration of all 4-node motifs extracted from a time series which only takes discrete values. Each motif can be characterized according to a hierarchy of inequalities in the associated time series.

The latter procedure is akin to defining a sliding window of size *p* in graph space that initially covers the first *p* nodes and sequentially slides, in such a way that, for each window, one can associate a motif by (only) considering the edges between the *p* nodes within that window, see Fig. 6 for an illustration. Not all possible graphs with *p* nodes are indeed sequential HVGm: an enumeration of low order motifs was provided in^12^, where it was shown that the lowest nontrivial order was $p=4$. In Fig. 7 we enumerate all possible motifs of order $p=4$ (only 6 possibilities). For the specific symbolization rule depicted in Table 1, we could outline a few examples of possible motifs present in the clickstream trajectories. For instance, the subsequence View-View-Detail-View (1121) is an example of a $Z_1$ motif, the subsequence Add-View-View-Remove (3114) is an example of a $Z_2$ motif, the subsequence Add-View-Add-Detail (3132) is an example of a $Z_3$ motif, and so on. We could be tempted to relate specific motifs to specific behavioral patterns. However, note that in the event the symbolization rule is changed (for instance, applying a permutation to the mapping in Table 1), then, different behavioral patterns would yield different motifs. This is true for all categorical sequences which need to undergo a symbolization process.

The simplest metric encapsulating statistics of the HVGm is the so-called profile **Z**, defined as the discrete marginal distribution of the set of HVGm. In this work we will only consider motifs of order 4, hence

$$\begin{aligned} \mathbf{Z}=[Z_1,Z_2,\dots ,Z_6], \end{aligned}$$

where $Z_i$ is the estimated probability of the appearance of motif *i*.

At this point, it’s important to recall that HVGm capture high-order patterns in the time series while being computationally efficient. As a matter of fact, in order to compute HVGm statistics, there is no need to extract the HVG from a time series and then partition the graph to detect motifs, as depicted in Fig. 6. There is an algorithmic recipe to look for HVGm directly in the time series in linear time^12^, provided that the detection of motif *i* in a trajectory reduces to checking for an inequality set, as shown in Table 7 for the case of motifs of order 4 extracted from discrete-valued sequences (see^12,23^ for additional technical details in the case where the sequence is real-valued). This aspect becomes key if one aims to implement this type of feature extraction method within an online application that predicts customer conversion in real time.

Hence, we consider $\mathbf{Z}$ as an additional predictor. Furthermore, in order to quantify the heterogeneity of a motif profile, we also compute the entropy of the HVGm profile:

$$\begin{aligned} h_z=-\sum _{i=1}^6 Z_i \log Z_i \end{aligned}$$

which is larger when the different motifs appear more evenly represented and lower when a particular motif is overrepresented.

### Trajectory length

**Figure 8:**
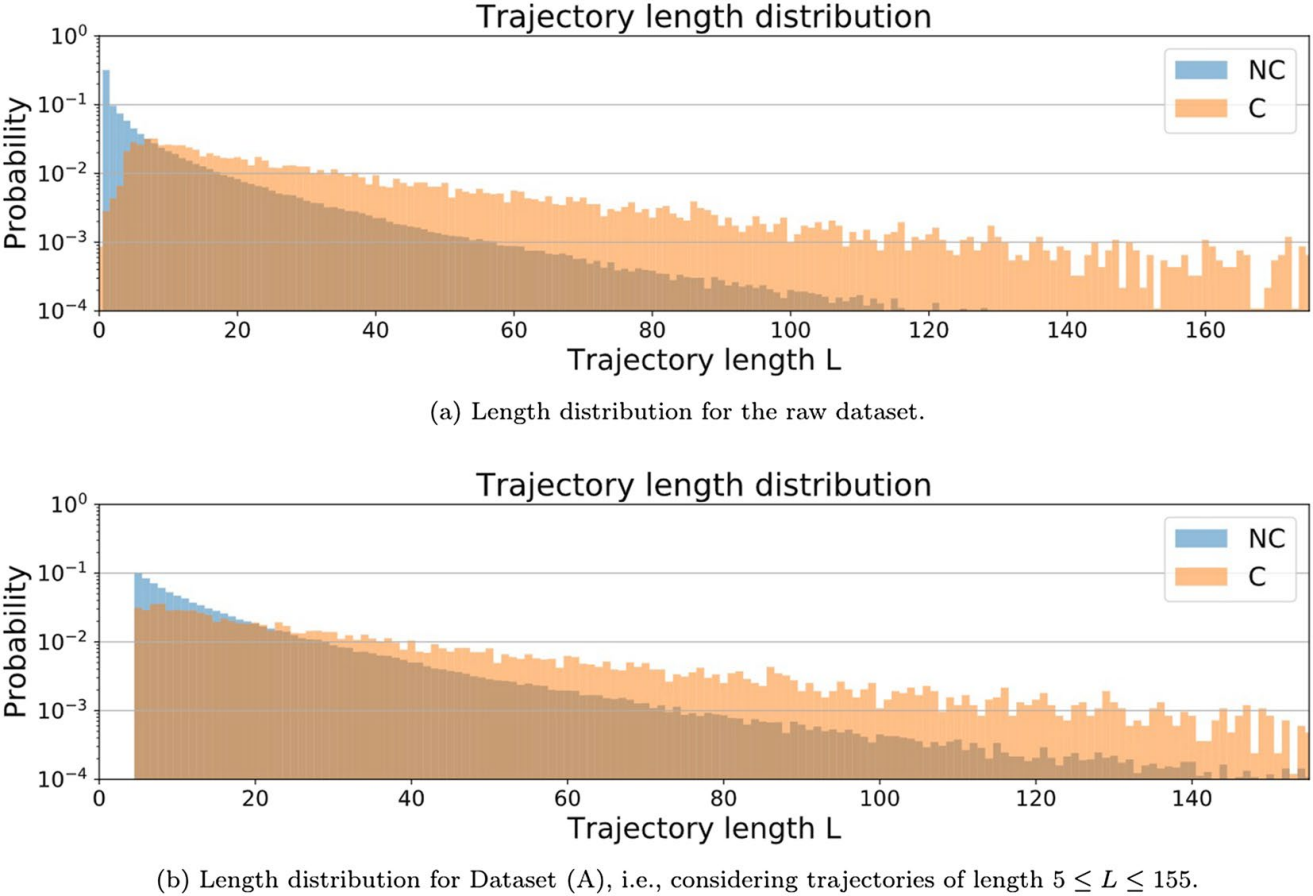
Trajectory length distribution for each of the classes.

One could argue that the length *L* of a given trajectory, defined as the number of individual clicks a user performs before leaving the session, could be indicative of the customer’s purchase intent. In Fig. 8 we depict the length frequency for the trajectories belonging to each class, where we can see that there are trajectories of all lengths in both cases. As both distributions are rather broad (especially for class C), the mean length is probably not a good indicator and any other information extracted would not be a discriminative feature for classification, provided that distributions heavily overlap. Note that, interestingly, there are more short trajectories belonging to the NC than the C class, despite the fact that the trajectories in the C class had been trimmed.

In any case, since our final goal is to perform early prediction—that is, being able to predict after only a few clicks whether a new customer will be eventually buying—we do not consider the trajectory length as a predictor in our classification analysis, as all trajectories have the same length in this scenario.

### Preface on classifier evaluation

In this section and the subsequent one (section "From hand-crafted to automatic feature extraction using neural networks"), we aim for a qualitative analysis of the tasks at hand, assessing the classification viability and what can be learned from the features. Hence, given that the datasets are heavily unbalanced (see section "Task-specific datasets"), in order to train and evaluate our classifiers much faster, we generate new subdatasets taking the totality of samples of the least represented class (C) and subsampling the same amount of samples from the majority class (NC). This way, both classes are perfectly balanced, which allows us to perform a qualitative analysis of the differences between them much more easily. We perform the benchmarking in a more realistic scenario in section "Benchmarking".

Then, we split the subdataset into train and test sets with a 80/20 ratio—we train the classifier on the train set and evaluate the accuracy on the test set. Furthermore, the classifier undergoes a 3-fold cross-validated (non-exhaustive) grid-search of its most relevant parameters during the training. As evaluation metrics, we use F1 score and area under the ROC curve, which we denote as AUC.

The whole subsampling procedure is repeated for 10 times and we report the mean value of the metrics together with their standard deviation over the repetitions.

### Purchase prediction

We first address the trajectory classification task using the Dataset (A) (see section "Task-specific datasets").

#### Feature importance

**Figure 9:**
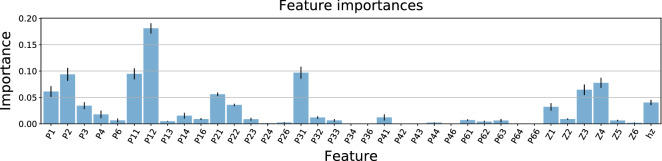
Feature importances of a XGBoost classifier. From left to right there are the 1-g and 2-g of the clickstream, and, then, there are the HVGms **Z** and their entropy $h_z$. For readability we have omitted the parenthesis in $P(s),\ P(s,s')$.

In order to have a preliminary idea of the relevance of the features that we have engineered, we train a XGBoost classifier^41^ over several balanced subdatasets and compute the feature permutation importances, as shown in Fig. 9. With this, we can obtain a better understanding of the underlying behavioural patterns that lead towards purchasing an item.

Given that symbol 3 is related to adding a product to the cart (recall Table 1), we would expect its sole presence *P*(3) to be quite significant. However, we find that it is rather the combination of two actions add-view (*P*(3, 1)) that is more relevant. Furthermore, this not even the most relevant feature, showing that the web exploration patterns, related with actions view and detail, actually provide the most important information. This is backed up by the importance of some HVGms, which show that some of these navigation patterns are, indeed, quite revealing. This is extremely important, provided that trajectories are mostly compound by these two actions, as shown in Fig. 4, meaning that most users jump from page to page (series of view (1)), product to product (series of detail (2)) and page to product (view-detail (12) or reverse) but rarely dive into a specific product straight from a search (click (6)).

**Figure 10:**
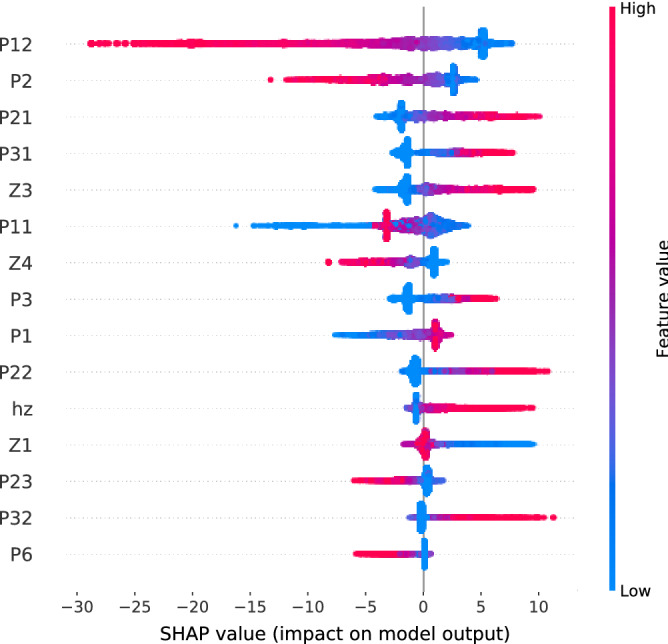
Influence of the top fifteen features in the classification of a balanced subsampling of the dataset (16,648 samples) with an XGBoost classifier. Each dot in the plot corresponds to a sample. On the y-axis features are listed in order of relevance, from top to bottom; on the x-axis the influence of the feature in the classification of the given sample is provided. The color codes the relative value of the feature for each given sample. Hence, the plot is read feature-wise, looking at how the different values of the feature contributed to the prediction of the samples: left is NC and right is C. Notice that the exact importance ordering may differ from the results in Fig. 9 within error bars.

In order to dive deeper into the analysis, we use SHAP’s tree interpreter^42,43^ to see the effect of each feature in the prediction. In Fig. 10 we see the influence of the most relevant features involved in the classification of a balanced sub-sampling of the dataset. In agreement with Fig. 9, the 2-g *P*(1, 2) corresponding to view-detail is the most relevant feature with high values indicating that the customer is, most likely, not buying before leaving. Combined with what we observe for the other features, behaviours such as wandering from page to page (high view-view *P*(1, 1)), opening many products (high view-detail *P*(1, 2) and detail *P*(2)) without going straight from one to another (low detail-detail *P*(2, 2)) and casually doing some searches (high click *P*(6)) are indicative that the customer at hand will not end up buying. High values of $Z_1$ and low values of the $h_z$ indicate that such trajectories are rather monotonic, which is not surprising when we think about a casual browser looking at what is offered. An example of a trajectory could be view-view-view-detail-view-detail (111212) providing high view-view (*P*(1, 1)), view-detail (*P*(1, 2)) and detail (*P*(2)), includes two $Z_1$ and a $Z_4$, producing a medium $h_z$. This example would correspond to a customer that scrolls through different pages until it finds a product to look at, goes back and then checks another product.

On the other hand, behaviours such as visiting few products and many different pages (high view *P*(1)) but coming from different actions (low view-view *P*(1, 1) and high detail-view *P*(2, 1), add-view *P*(3, 1)) are indicative that the user will purchase an item before leaving. Other recognisable patterns involve going straight from one product to another (high detail-detail *P*(2, 2)), which suggests that these users may take advantage of features such as similar product recommendations. High values of the HVGm entropy $h_z$ indicate that there occur many more different navigation patterns and, hence, the browsing activity is richer in terms of the number of different actions. An example of a trajectory with such properties could be view-add-detail-view-add-view (132131) with low detail *P*(2) and view-view *P*(1, 1), but with high view *P*(1) and add *P*(3), as well as add-view *P*(3, 1) and detail-view *P*(2, 1). It contains a $Z_1$, $Z_3$ and $Z_5$, maximizing the pattern entropy $h_z$. Overall, this is indicative that, in general, customers that end up buying tend to have a prior idea of what they want.

It remains to be explored whether different symbolizations from the one in Table 1 may highlight different behavioural patterns in terms of the HVGms $\mathbf{Z}$. $Z_1$ is the motif that contains the most possible patterns (see Fig. 7) and it is the most common. Other HVGms are more restrictive and represent more specific scenarios, such as $Z_3$ (indicative of class C) and $Z_4$ (indicative of class NC). With other symbolizations, patterns that are currently compressed in $Z_1$ may fall into other motifs, which might turn out to be characteristic of one class or another.

#### Pipeline and evaluation

We then proceed to build the pipelines for each of the five classifiers we consider, namely (1) a logistic regression (LR) classifier, (2) a random forest classifier (RF)^44^, (3) a support vector classifier (SVC), (4) a XGBoost classifier^41^, and (5) a shallow dense neural network (NN). Each pipeline originally contained (1) a pre-processing part, where we explored using two different types of dimensionality reduction: principal component analysis (PCA)^45^ and uniform manifold approximation and projection (UMAP)^46^, subsequently followed by (2) the classification part, with the specific classifier. In the case of the NN we include an intermediate feature normalization step subtracting the mean and dividing by the standard deviation.

The pipelines are evaluated as previously explained in section "Preface on classifier evaluation". Interestingly, we have found that it is best to refrain from performing any dimensionality reduction, provided that the features are fairly compact already, as (1) adding the pre-processing impacts the performance negatively (between 1% to 3% drop in F1 depending on the methods) and (2) removing dimensionality reduction does not compromise results, e.g. no overfitting takes place, as shown below. This is quite relevant, as it removes a significant computational workload and makes the whole pipeline considerably faster. Hence, we simply apply the classifiers to the features extracted from the data and obtain the results reported in Table 2. The best classifiers output F1 scores around $87-88\%$. The NN holds a significant advatange in terms of AUC with respect to the rest, suggesting that it has a much better notion of separability between the two classes and that most errors come from overlapping samples of different classes. Note that since, in principle, the HVGm profile depends on the symbolization rule, higher scores could, in theory, be achieved using a different rule that enhances the discrimination between classes. Given that we lack a direct way of finding such optimal configuration a priori, we simply consider the obtained classification scores as a lower bound, and we leave the general task of finding the optimal symbolization rule as an open problem for future research.

**Table 2:** Mean F1 score and area under the ROC curve over 10 subsamplings of the dataset ± standard deviation for the different classifiers.

Classifier	F1 (%)	AUC (%)
LR	$84.05 \pm 0.61$	$84.67 \pm 0.52$
RF	$87.65 \pm 0.84$	$87.70 \pm 0.82$
SVC	$87.46 \pm 0.40$	$87.65 \pm 0.38$
XGB	$88.08 \pm 0.39$	$88.17 \pm 0.35$
NN	$\mathbf{88.17 \pm 0.61}$	$\mathbf{94.53 \pm 0.28}$

**Figure 11:**
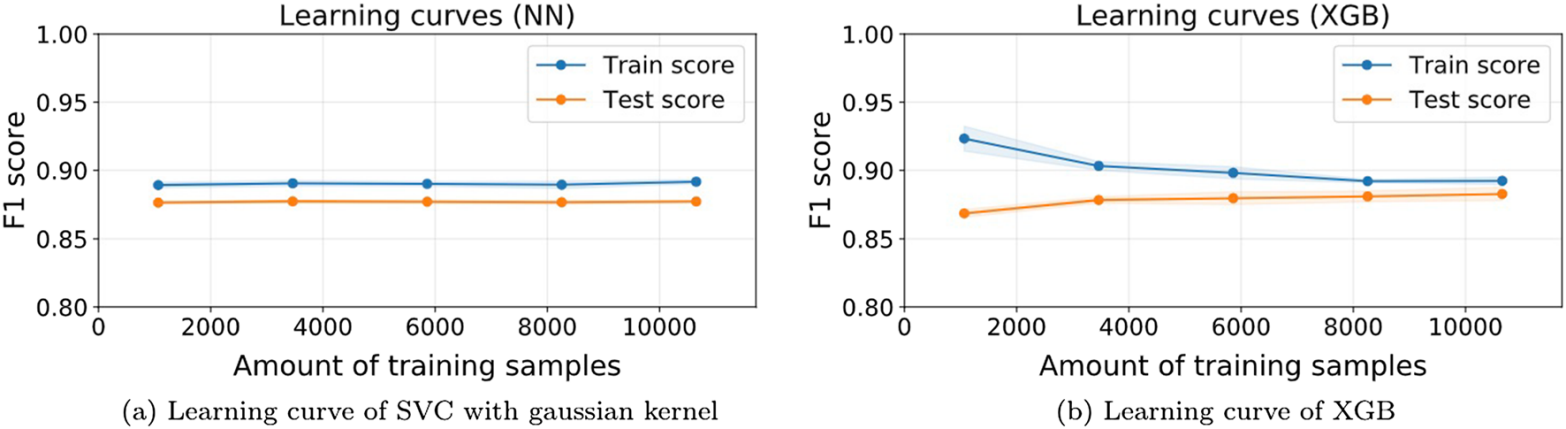
Learning curves examples of two of the best performing classifiers: (**a**) SVC and (**b**) XGB. The models are evaluated through nested cross-validation of 5 outer folds and 3 inner folds to optimize the hyper-parameters of each model through a grid-search in balanced sub-datasets of limited amount of samples. The curves depict the mean accuracy over the 5 folds and the shaded region represents its standard deviation for both the training and test sets as function of the amount of data used.

#### Learning curves

As an additional test, we also look at how the best classifiers behave with increasing data. Indeed, in any industrial application, the database is continuously growing as new customers browse the online store, making it essential to us to ensure that whatever method we choose to implement will behave properly in a near future and, even more importantly, see whether it can improve its performance with further data.

Figure 11 depicts the learning curves of two of the best performing classifiers: NN (panel a) and XGB (panel b), based on the results in Table 2. We can see how the NN performance seems to be significantly unaltered with respect to the amount of training data, keeping a constant gap between train and test scores. This means that with little amount of samples the classifier can already perform competitively.

In contrast, the XGB classifier seems to be continuously improving with further amounts of data. However, in the grand scheme of things, the amount of data does not have a massive impact beyond $\sim 6000$ samples. Hence, even though we see that the classifiers could potentially improve with further samples, the results will not be much different from what can be found in Table 2. As a final remark, we can also see in Fig. 11 that the classifiers are not overfitting despite not using any kind of dimensionality reduction.

### Early prediction

Now that we have found that features are informative and that conversion is highly predictable, in a second step, we are interested in the notion of early predictability, i.e. the capacity to predict that a new user will eventually purchase an item by only looking at the first *T* datapoints of the trajectories. For this, we now work with Dataset (B) (see section "Task-specific datasets"). Since our set of features is intrinsically scalable for large datasets (e.g. HVGm is extracted in linear time), finding evidence of early prediction ($T\ll L$) opens up the possibility of implementing prediction algorithms online for a real-time assessment of a customer conversion probability. This finding would prove to be highly significant for e-commerce technologies as individual tuning of marketing strategies would then be a realistic possibility.

To assess early predictability, we have designed the following experiment. We vary the early window *T* in the range $T=5,\dots ,14$ and, for each value of *T*, we perform the following steps:

- First, we extract the corresponding sub-dataset from our pre-processed Dataset (B), as explained in section "Task-specific datasets". So, effectively, we are only considering the initial part of each trajectory (the first *T* datapoints).
- Then, we perform binary classification as performed in section "Purchase prediction", considering the same range of introduced classifiers.
- Finally, we compute an earliness parameter defined as
  $$\begin{aligned} \text {Earliness}=100\bigg [1- \frac{T}{\Omega }\sum _{i=1}^{\Omega } \frac{1}{L_i}\bigg ], \end{aligned}$$
  where $L_i$ is the size of the *i*th trajectory and $\Omega$ is the total number of trajectories. This quantity provides an idea of ’how early’ a trajectory is predicted with respect to its length *L*, i.e. in the case of the C class, how much in advance of the first purchase event. When the early observation window *T* is large, then $\text {Earliness}\rightarrow 0\%$, and as the window shrinks the earliness parameter increases (the limit value depends on the actual size of all trajectories $L_i$). The earliness provides, on average, the relative distance between the point at which the prediction is performed with respect to the end of the trajectory. For example, considering a window of length $T=5$, a trajectory of length $L=5$ will have an earliness of $0\%$, while a trajectory of length $L=10$ will have an earliness of $50\%$ and a trajectory of length $L=20$ will have an earliness of $75\%$.

**Figure 12:**
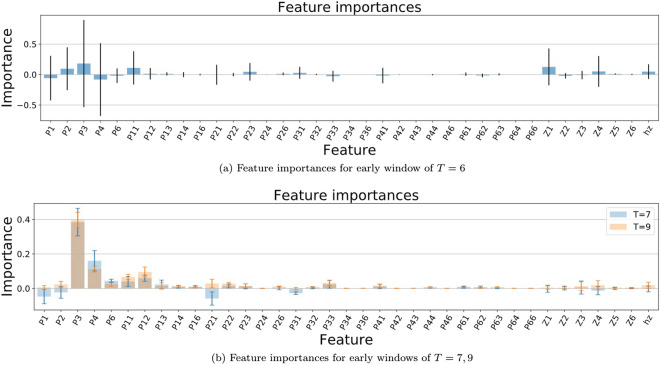
Permutation feature importances of the XGB classifier for the early detection problem, at different values of the early window size: (**a**) $T=6$ and (**b**) $T=7, 9$. The features are the same as those explained in Fig. 9. For short early windows, the classifier is confused by some features and, as the early window grows, it obtains a better understanding.

Before addressing the classification results, we run a feature analysis, shown in Fig. 12, similar to the one performed in section "Purchase prediction", for various values of the early window $T=6,7,9$. For very short time windows (panel a), the relevance of the features varies significantly between repetitions and the XGB classifier is confused by some of them such as *P*(1) and *P*(4). As the observation window grows, the variance between repetitions is significantly cut (notice the major differences between $T=6$ and 7) and the confusion is reduced up to the point that, at $T=9$ (panel b), there are no harmful features (no negative importance). For such short early windows, we observe that the most relevant features are the presence of Add (*P*(3)) and Remove (*P*(4)) actions. The classifiers tend to be more naive, given the reduced amount of navigation information, and focus on the early addition and removal of products to the cart, in contrast to what we observed in the full trajectory classification (Fig. 9).

**Figure 13:**
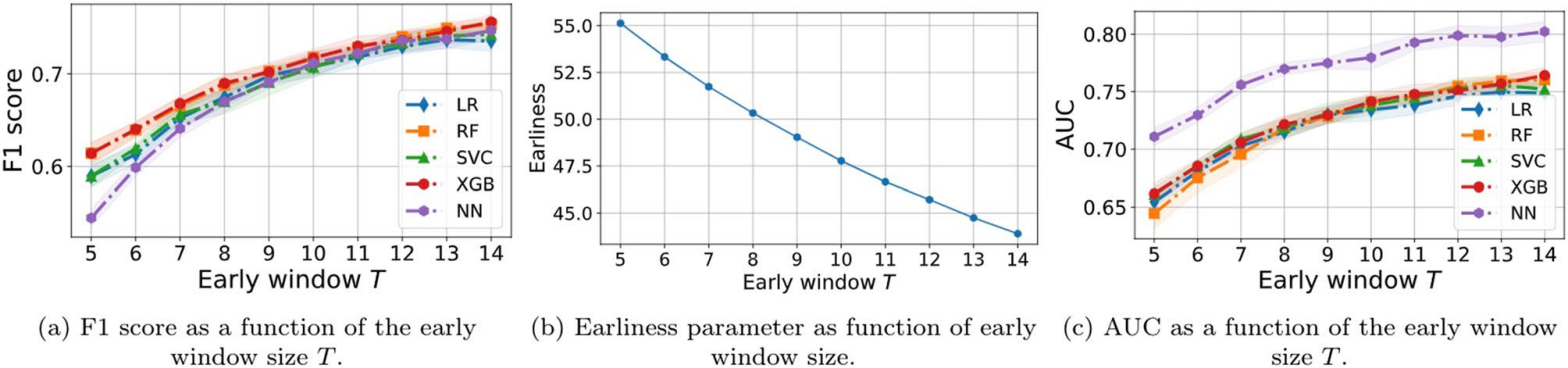
(**a**) F1 score curves of the different classifiers as function of the early window size *T*, where only the first *T* datapoints of each trajectory are used to extract the features. (**b**) Earliness parameter of the sub-datasets generated for each early window *T* (recall section "Task-specific datasets"). (**c**) AUC as a function of the early window size *T*. Results indicate that reliable early prediction of conversion is indeed possible.

The classification results are then summarized in Fig. 13, where we find that early prediction of customer intent is indeed possible, reaching F1 scores beyond 60% for $T=5$, and increasing beyond 70% from $T=9$ onward (panel a). Looking at the earliness, shown in panel b of the same figure, these predictions are made with around $50\%$ anticipation, meaning that, on average, the actual live sessions are more than twice as long, hence allowing the system for some extra time to implement personalisation strategies. This is also the main reason behind the change in feature importance with respect to full trajectory classification. In panel c of the same figure we can see the difference in AUC of the NN with respect to the rest of the classifiers, which, interestingly, provides the worst F1 for the shortest windows.

## From hand-crafted to automatic feature extraction using neural networks

To complement the previous analysis, we also consider two DL approaches using a simple Markov Chain as a baseline. The first one implements a generative model, replacing the Markov Chain with a Long Short Term Memory (LSTM) network. The network is trained using an auto-regressive loss function, which captures how well the model predicts the next symbol in a sequence. The second approach implements a discriminative model, also using a LSTM network, which is in this case trained to predict the sequence class.

### Markov chain classifier (MC)

As is often recognized in the literature^3,4^, there are strong resemblances between the clickstream prediction problem and standard topics in the Natural Language Processing (NLP) community, as many NLP-tasks are (1) sequential and (2) based on a discrete set of atomic states. As our general baseline, we pick a *k*-gram Markov Chain, a popular choice for language modeling, at least before the DL revolution. Clickstream sequences are, first, separated by class, given that a Markov Chain is trained for each class. Then, the trajectories are featurized extracting *k*-grams. A transition matrix is created by counting how often the last click in a *k*-gram co-occurs with the preceding clicks in the same *k*-gram. Co-occurrence frequency counts are then normalized to obtain conditional probabilities.

At inference time, trajectories are classified according to Bayes rule. Consider the conversion class C. The probability of finding this class *p*(*C*) in the training corpus is the *prior*, whereas the conditional probability of observing a given trajectory ${{\mathcal {S}}}$ under the Markov Chain trained on C sequences is the likelihood $p({{\mathcal {S}}}| C)$. Analogously, the conditional probability that the trajectory belongs to the NC class is $p({{\mathcal {S}}}| NC)$. Hence, the posterior reads

1 $$\begin{aligned} p(C|{{\mathcal {S}}}) = \frac{p({{\mathcal {S}}}|C)p(C)}{p({{\mathcal {S}}}|C)p(C) + p({{\mathcal {S}}}|NC)p(NC) } \end{aligned}$$

We set a classification threshold $t=0.5$ such that trajectories are classified as C whenever $P(C|{{\mathcal {S}}}) > t$, and as NC otherwise. Essentially, we derive two generative models: one for positive and one for negative clickstreams. The classification is done by running the trajectories through both Markov Chains and predicting the class corresponding to the Markov Chain which is least surprised, i.e. the one that assigns a higher probability weighted by the priors. We explore *k*-grams with $2\le k\le 5$ and, unsurprisingly, we find that 5-g provide the best results. Finally, since the prior probability for each class changes as a function of the sequence length, we compute length-sensitive priors dividing the number of C trajectories of a certain length by the total number of trajectories of the same length. In order to prevent sparsity, we bin lengths by one up until $L\le 50$, by ten for lengths $50 < L \le 100$ and by twenty-five for lengths $100 < L \le 150$. Lengths longer than $L\ge 150$ fall into the same bin.

### LSTM generative classifier (LM LSTM)

A recent paper by the technology team of a leading Japanese e-commerce website^4^ reported improvements over the MC approach in^3^ using LSTMs. While they frame the problem as a three-fold classification (purchase, abandon or browsing-only), they use the same idea of “mixture models” as in^3^ only replacing Markov Chains with probabilities from a neural network model (token probabilities are read off intermediate softmax layers in each LSTM model). We build two LSTMs, one for each class, with as many input and output units as possible actions (Table 1) plus a beginning and end of sequence symbol—one hot encoded. In line with^4^, we consider architectures with one hidden layer, such that between the input and output layers, we include one LSTM layer of size (input units $\times$ hidden units) and one fully connected layer of size (hidden units $\times$ output units). However, unlike^4^, we do not consider models with 2 hidden layers and do not explore the effect of dropout. We use Cross Entropy as our loss function and train the network using an Adam optimizer.

At inference time, trajectories are passed through both LSTMs retrieving the the probabilities of every state in the trajectory. The prediction is performed in a Bayesian way, using Eq. (1). Again, the prior is length-sensitive, computed in the same way described for the Markov Chain.

### LSTM discriminative classifier (S2L)

Building on top of the generative LSTM (LM LSTM), we implement a discriminative classifier as an alternative way to conceptualize the clickstream problem. Training happens by feeding batches of trajectories and training the model to minimize prediction error to the binary output class. This architecture consists of one LSTM layer of size (input units $\times$ hidden units) and one fully connected layer of size (hidden units $\times 1$), whose output is transformed with a sigmoid activation function.

We explore two classification strategies changing the information that is fed into the fully connected layer from the LSTM layer. We consider two pooling strategies: taking the LSTM output at the last time step (S2L last) and taking the average LSTM output over the whole trajectory (S2L avg). We use Binary Cross Entropy as the loss function and Adam as the optimizer.

### Hyper-parameter grid search

We start by performing a full grid search on full trajectory prediction. Each model is trained on 70% of dataset (A), and the remaining sequences are evenly split into validation and test sets. The procedure is repeated 10 times to account for the stochasticity in the downsampling step, thus providing an average over 10 different subdatasets.

The different models are trained using early stopping for a maximum of 50 epochs with a patience of 10 taking the loss on the validation set as the target variable. Notice that the Markov Chain classifier does not use an optimizer and is simply trained by estimating the transition matrices.

For the Markov Chain classifier we consider *k*-grams with $k=2,\dots ,5$. We consider a simple Laplace smoothing and a trivial back-off model without discounting and find that the former performs better. Other, more elaborate, smoothing techniques are unlikely to yield substantially different results given the simplicity of the transition matrix. For the DL models, we explore different sizes of the hidden layer (16, 32, 64, 128), batch sizes (16, 32, 64) and learning rates (0.01, 0.001). We perform an exhaustive grid search for full trajectory classification.

Having identified the best hyper-parameters for the full trajectory classification, we run a partial grid search for the early prediction problem. The best performing parametrization on the full sequence prediction is included along with three other parametrizations to see whether different architectures yield remarkably different results on trimmed sequences. For the Markov Chain classifier we consider one other order next to the one which yielded the best result on whole trajectory classification.

In general, grid searches do not reveal strong differences across parametrizations, especially for short early windows. Small differences emerge at longer windows and on whole trajectories, but they are unlikely to reflect in significant performance changes. Thus, these methods are rather robust to the choice of hyper-parameters. The best performing hyper-parameters implemented thereafter for each model are:

**Markov Chain:** order 5 with Laplace smoothing.
**LM LSTM:** 64 hidden units, 32 trajectories per batch and learning rate of 0.001.
**S2L (avg):** 64 hidden units, 16 trajectories per batch and learning rate of 0.01.
**S2L (last):** 32 hidden units, 16 trajectories per batch and learning rate of 0.001.

### Results

#### Full trajectories

Classification results evaluated on the whole trajectories are reported in Table 3. Overall, there is a performance improvement with respect to the feature engineering results reported in Table 2. The Markov chain classifier lags behind with the network implementing last pooling holding the best performance. The generative methods, i.e. the Markov chain classifier and the LM LSTM, underperform with respect to the discriminative models in all aspects.

**Table 3:** Mean F1 score and AUC ± a standard deviation over 10 subsamplings of the dataset for the different classifiers.

Classifier	F1 (%)	AUC (%)
Markov ($k=5$)	$89.45 \pm 0.61$	$95.51 \pm 0.45$
LM LSTM	$90.21 \pm 0.36$	$96.01 \pm 0.36$
S2L (avg)	$90.89 \pm 0.49$	$96.44 \pm 0.34$
S2L (last)	$\mathbf{91.03} \pm \mathbf{0.48}$	$\mathbf{96.83} \pm \mathbf{0.37}$

**Figure 14:**
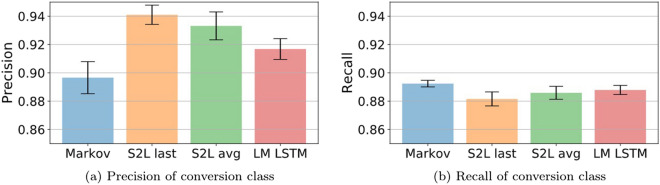
Classification performance analysis for full sequences considering: (**a**) precision for the conversion class and (**b**) recall for the same class. Patterns in precision and recall for the non-conversion class mirror patterns in recall and precision of the C class, respectively.

We deepen into the analysis by looking at the precision and recall, depicted in Fig. 14, for the C class (Keep in mind that, with a balanced test set and two output classes, C and NC, precision on NC sequences mirrors recall on C sequences while recall on NC sequences mirrors precision on C sequences. For this reason we only show precision and recall for C sequences). The discriminative models hold better precision, while the generative models pull ahead slightly in recall. Nevertheless, the recall is rather consistent among all the different models, but we can see substantial differences in the precision. Hence, these differences in precision are the main cause of the differences in overall performance. We can see that the disadvantage of the Markov Chain classifier comes from its significantly inferior precision, despite holding the best recall among classifiers. On the other hand, the S2L with last pooling holds the best precision by far, while having the worst recall. The performance of the discriminative LSTMs is quite similar, with the last pooling showing an edge in precision at the cost of some recall, where the average pooling takes the lead. Nevertheless, the gain in precision is higher, thus bringing the S2L with last pooling ahead, as shown in Table 3.

#### Early prediction

**Figure 15:**
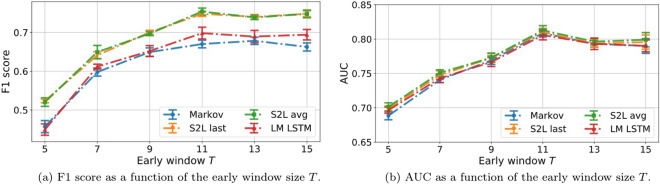
(**a**) F1 score and (**b**) AUC as function of the early window size for early prediction. These models provide worse F1 scores than the feature engineering ones in Fig. 13.

We then move on to early prediction, where, as shown in Fig. 15 the results are comparable to the ones obtained with handcrafted features shown in Fig. 13. We observe that the models based on feature engineering outperform, in terms of F1 score (panel a), all the models introduced in this section in both short and long early windows. Only the discriminative LSTMs achieve similar performance for long windows. In terms of AUC, the DL-based models have the advantage, although the shallow neural network feeding on the features achieves the same AUC or arguably better as we discuss below (panel b).

As in the full trajectory classification, the generative models are outperformed by the discriminative ones. As before, the Markov Chain performs the worst, but, this time, both S2L with average and last pooling provide the same results. All models have similar AUC with meaningful differences only emerging for $T>13$. Interestingly, the curves are non-monotonic and peak at $T=11$, exhibiting a slightly decreasing trend thereafter. It seems counter-intuitive that providing the models with more information results in lower performance. One possible explanation may reside in the size of the training data, which is reduced as the early window increases, since only trajectories of length $L\ge T$ are considered in each subdataset. The observed behaviour may be explained by the combination of two factors: increasing trajectory information and decreasing training samples. This is not observed in the feature-engineering models, which exhibit a clear monotonic tendency (Fig. 13) and are robust to the dataset size (Fig. 11), which is why the AUC obtained by the NN classifier is arguably better.

**Figure 16:**
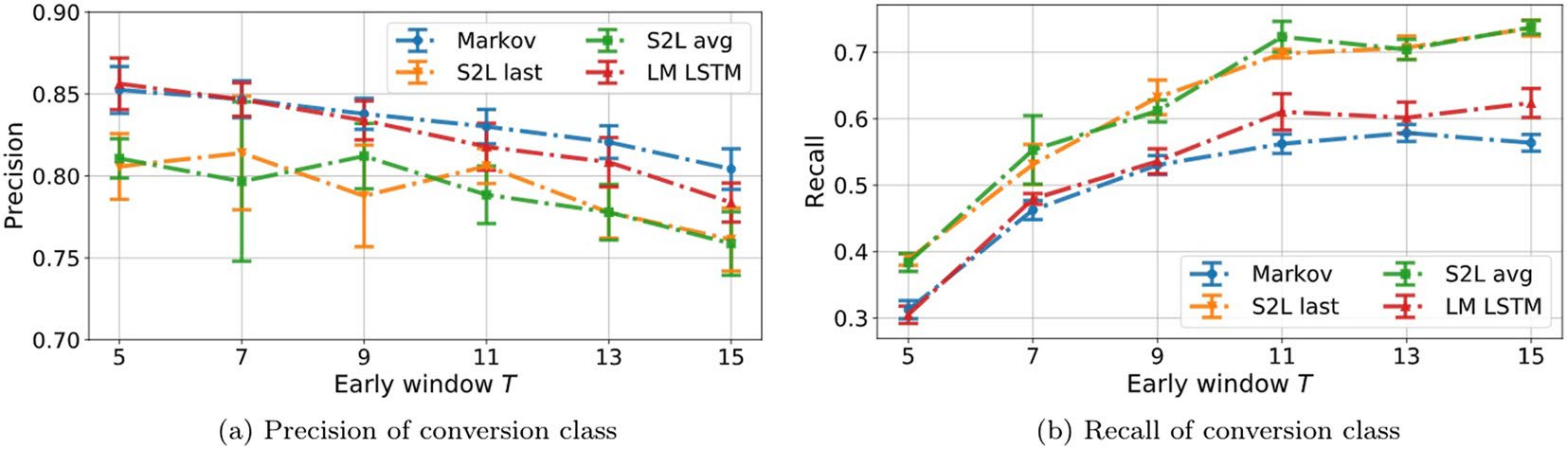
(**a**) Precision and (**b**) recall of the conversion class as function of the early window size.

Diving into precision and recall analysis, we see a consistent pattern over time. Classifiers become less and less precise in classifying sequences which include a *purchase* event. The pattern for recall follows the opposite trend, as classifiers tend to be more reliable. In contrast to what we observed in the task of full sequence classification (Fig. 14), the generative models exhibit higher precision and lower recall than the discriminative models.

To summarize, we observe a reliable edge for discriminative over generative classifiers in both prediction tasks. In early prediction, performance increases with more available clicks up to $T=11$. The overall pattern arises from a decreasing precision on C trajectories and an increase in recall over the same class. This suggests that, at early stages, classifiers tend to predict the NC class, resulting in a higher precision for C sequences at the cost of mistaking several of them for NC. Widening the early window, this trend weakens and classifiers guess both classes more evenly; there is a reduction in precision for the C class, meaning that some sequences predicted to end in a *purchase* event are mislabeled, but there is also a much stronger increase in recall (note the range of the y axis in the two subplots in Fig. 16), meaning more actual C sequences are correctly identified.

#### Error analysis

In order to dig deeper in the behaviour of the classifiers and to analyse how certain general properties of a sequence, such as its true class and its length, interact with classification performance, we perform an error analysis. For each trajectory, we record its original length before trimming, its true class and the class that is guessed by each classifier. Then, we fit simple logistic regressions predicting classification accuracy as a function of the interaction between trajectory length, true class and classifier. When analyzing the early prediction setting, we also include the early window size (only considering $T=5$ and $T=15$) as an additional covariate.

**Figure 17:**
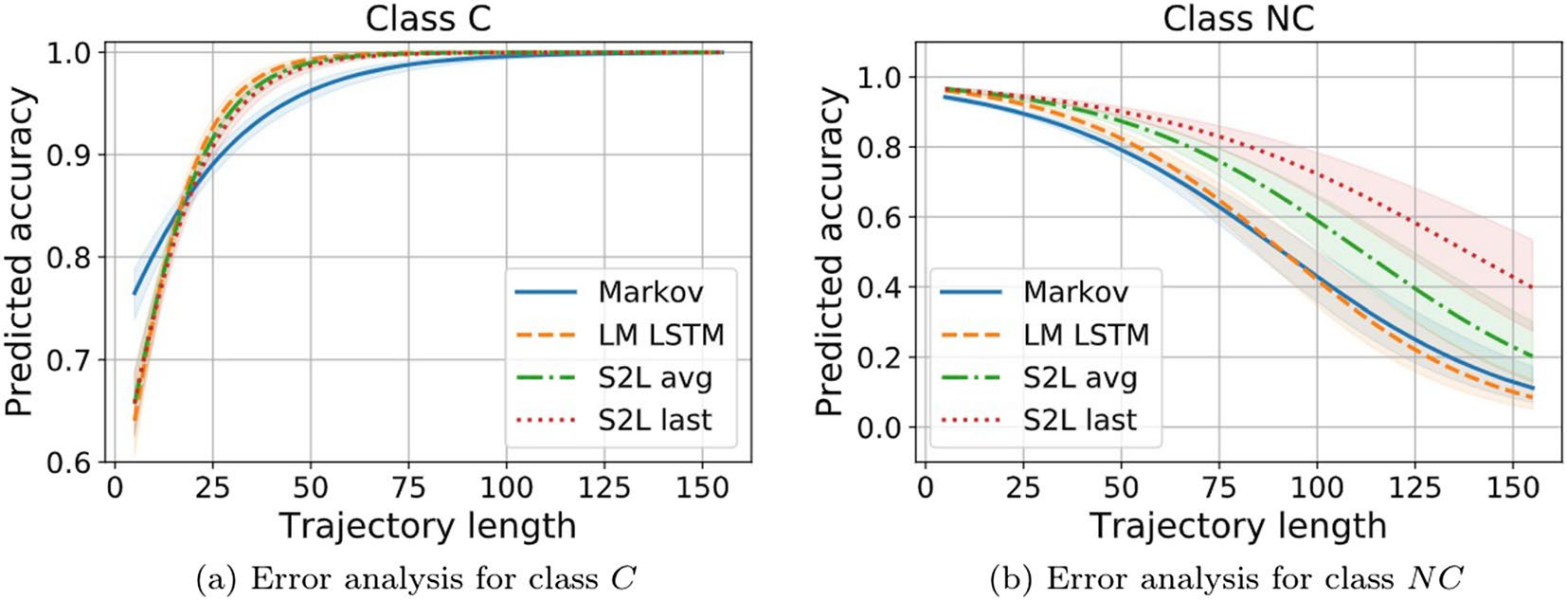
Predicted accuracy (y axis) as a function of the interaction of trajectory length (x axis), true class: C (**a**) and NC (**b**), and classifier (colour legend). The curves illustrate how the probability of correctly classifying a sequence changes as a function of the three covariates.

Figure 17 shows the outcome of this analysis for the classification of whole sequences. The y-axis indicates the predicted probability of correctly classifying a trajectory as a function of its true label, its length and the classifier. Starting with the *conversion* class, we observe that all the models correctly classify long sequences. In general, they struggle with short trajectories, with the Markov chain classifier being the most resilient one, but then having the worst scaling with length. Moving on to *non-conversion* sequences, we observe a similar trend in all models that is the opposite of what we observe with the *conversion* ones: short trajectories are perfectly classified while longer ones are not. The discriminative models are the most resilient to trajectory length, with the S2L with average pooling doing best, while the generative ones suffer the most with long trajectories.

This suggests that short trajectories tend to be classified as NC while longer ones tend to be classified as C. It makes sense, provided that, as seen in Fig. 8, the shortest trajectories tend to be from customers that do not end up buying, while longer ones tend to end up in a purchase event. However, this could also mean that the behavioural patterns conducting to a purchase event grasped by the models tend to be long and tend to appear in longer sequences regardless of the outcome. Crucially, we observe small differences between models for trajectories shorter than 25 clicks, which is where the bulk of the data lies (see Fig. 8), indicating that the overall model performance for the more common sequences is largely comparable, in spite of their very different complexity.

**Figure 18:**
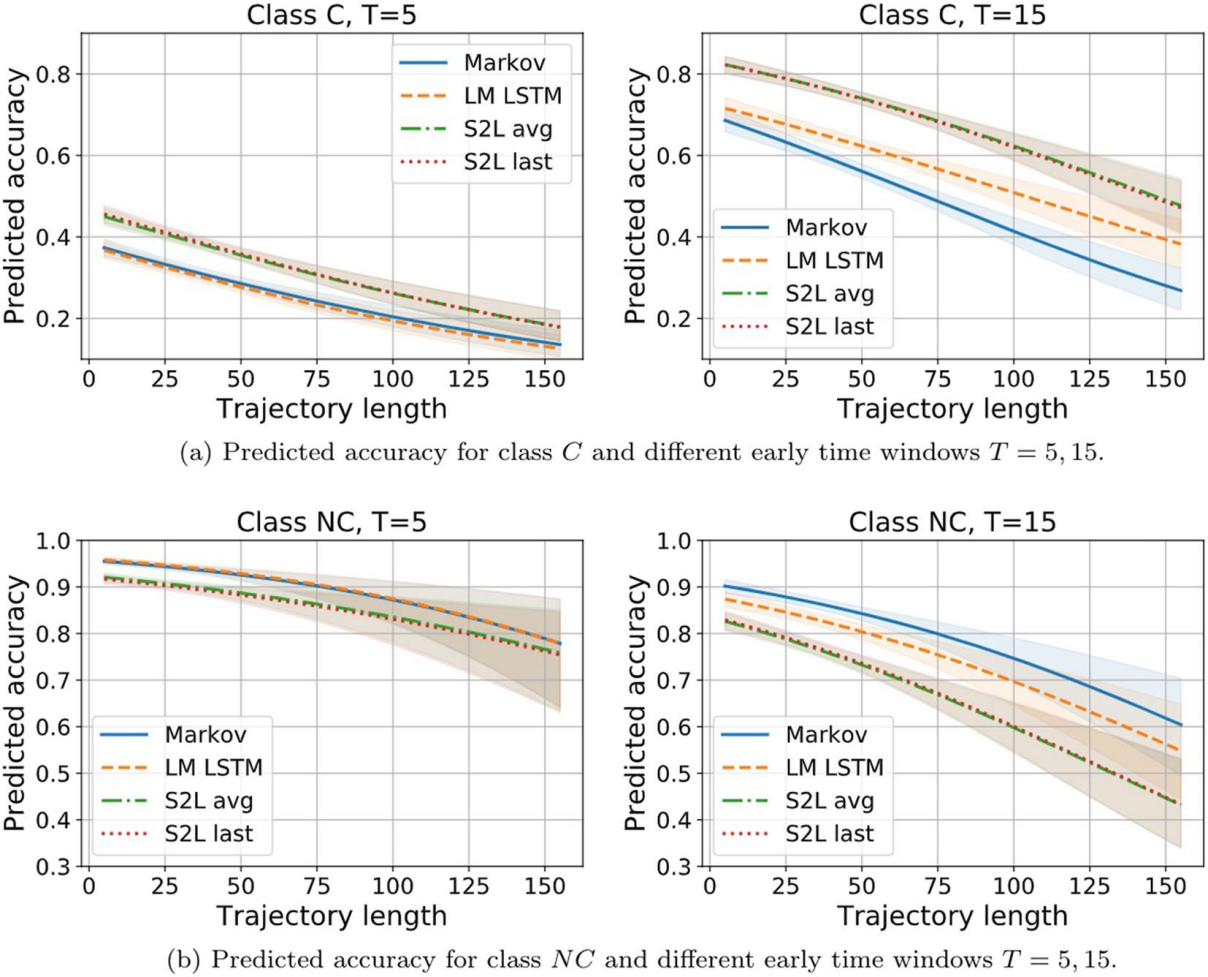
Accuracy (y axis) as a function of the interaction of sequence length (x axis), sequence true class (C and NC for (**a**) and (**b**), respectively), early window size $T=5, 15$ (columns) and classifier (color legend). The curves show how the probability of correctly classifying a sequence changes as a function of the four covariates.

Figure 18 shows the outcome of the same analysis performed for the early prediction task and, therefore, it accounts for the early window size. We observe that, while the accuracy increases as a function of the early window size for the conversion class, it decreases for the non-conversion one. Nonetheless, the improvement in accuracy for the C trajectories is much more significant than the drop in the NC trajectories. Therefore, the accuracy improves with early window size in agreement with what is shown in the previous section (Figs. 15 and 16). Again, all classifiers struggle with long NC sequences, but it seems that classifiers trained on the shortest early windows provide better results. Nonetheless, this time, all classifiers struggle with long trajectories also for the C class and, particularly, in short window sizes.

This analysis mostly confirms observations and other intuitions previously discussed. For example, it is hardly surprising that, by exposing the classifiers to fewer clicks per trajectory, they become less able to correctly classify them, provided that the information about the user intent might occur later on or it might be found in long distance dependencies. However, the analysis helps to highlight the most difficult task at hand, that is, properly identifying customers that will end up purchasing with very few clicks, as shown in panels a. The results from Fig. 18 indicate that, when the models only have access to the very initial clicks of the trajectory, they tend to classify them as NC. This is in line with the previous analysis in full trajectory classification from Fig. 17, where short trajectories tend to be predicted as NC, thus suggesting that the information about customer intent tends to be at the end and not at the beginning of the trajectories.

## Benchmarking

In this section, we evaluate our models with a more realistic approach in which we consider several sub-datasets with different class balances that range from the 50:50 (C:NC proportion) balance shown in the previous sections to the original dataset balance of $\sim 4:96$. This way, we cover a wide spectrum of cases, provided that class balance may be a major determinant of model performance and the one found in our application may not be representative of other websites. Unlike in previous simulations, however, models are trained with whole trajectories and we have them perform both early prediction and full sequence prediction tasks. This procedure aims to replicate the requirement that would be involved in a real business setting, in which a single model is asked to make predictions at different time steps.

To generate the different scenarios, we first split the original dataset into train and evaluation sets, with proportions of 70% and 30%, respectively. The test set is further split evenly into two sub-sets: a validation set for early stopping of the DL models and a test set used to assess the model performance. Then, we down-sample the majority class to obtain the desired class balance in the validation and test sets, since what matters is how the imbalance at test impacts performance. We perform ten repetitions of the whole process in order to account for the stochasticity in the down-sampling and splitting processes and the models are trained and evaluated in the same data without hyper-parameter fine-tuning.

**Figure 19:**
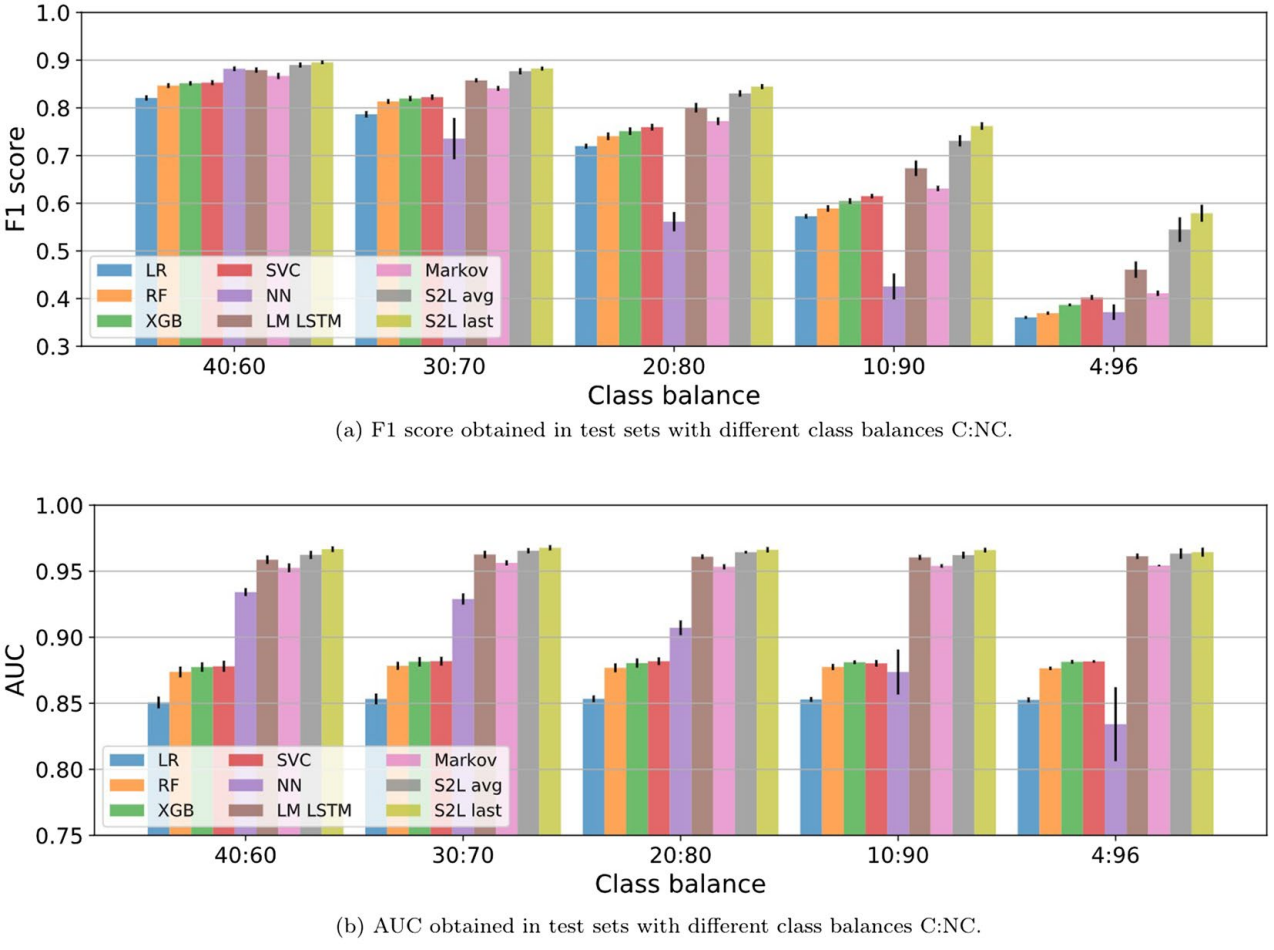
(**a**) F1 and (**b**) AUC results on model benchmarking in more realistic scenario. All models are trained on balanced datasets and evaluated on test sets with different C:NC proportions.

We opt for downsampling the majority class to match the sample size of the minority class in the training set. We observed that resampling the training set yielded better performances than when using the original dataset with a strong class imbalance. Moreover, we did not notice any sizable difference between downsampling and upsampling and preferred downsampling in order to speed up the computations. This way, the models are trained on balanced training sets and evaluated on test sets with different class balances. The down-sampling makes a major difference in the training time of the DL-based models and the SVC. In a sense, we assess how the models introduced in the previous sections behave in more realistic scenarios, changing the class imbalance at test time to show how different models scale. Figure 19 shows the obtained results on full trajectory classification and Fig. 21 shows the results on early prediction.

Focusing on full trajectory classification (Fig. 19), the S2L model with last pooling proves to have the best performance, in terms of F1 and AUC, while the LR classifier consistently performs worst, meaning that this classifier is not able to fully capture the relevant non-linearities in the data. The NN classifier suffers the most from class imbalance in terms of F1. However, the discrepancy between F1 and AUC suggests that it is most likely an issue related to the classification threshold not being optimized for the test imbalance. Seeing this, we train the feature-based classifiers with the original class balance without down-sampling the training set and, instead, we opt for a class re-weighting strategy. With this, we significantly improve the performance, as shown in Table 4, by more than 10% F1 for the XGB and NN classifiers, which now outperform the generative DL-based models and the RF matches the performance of the LM LSTM.

**Table 4:** Mean F1 score and AUC over 10 train/test partitions of Dataset (A) ± standard deviation.

Classifier	F1 (%)	AUC (%)
LR	$40.41 \pm 0.50$	$77.43 \pm 1.29$
RF	$45.77 \pm 0.63$	$73.07 \pm 1.84$
XGB	$49.87 \pm 0.58$	$79.87 \pm 0.84$
NN	$\mathbf{50.78} \pm \mathbf{0.27}$	$\mathbf{94.75} \pm \mathbf{0.07}$

The models are trained and evaluated with the original class balance of 4:96 following a class re-weighting strategy instead of downsampling (Fig. 19). We have refrained from training the SVC as it was extremely costly for such amount of samples.

Overall, the DL-based models outperform the feature-engineering-based ones. For mild class imbalance, the performance in terms of F1 is comparable among all methods and differences increase with further imbalance. Among the feature engineering models, the NN and XGB are the best performing classifiers, with the NN outperforming the LM LSTM and the Markov chain classifier at low imbalance. This shows that the HVGm features improve a simple 2-g feature representation to the point that it outperforms a statistical language model that leverages transition probabilities in *k*-grams up to $k=5$. Among the DL models, the discriminative methods outperform the rest, with the S2L with last pooling being the best.

**Figure 20:**
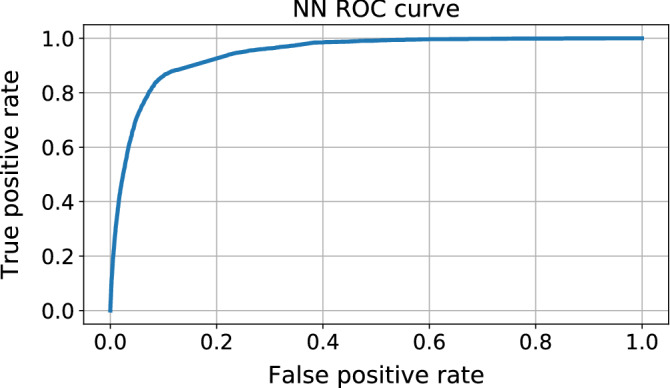
ROC curve of the NN classifier trained with the original class imbalance. The heavy imbalance of proportion $\sim$4:96 (C:NC) is handled through classes re-weighting with weights 2.5 and 1 for the C and NC class, respectively.

The fact that all models keep a high and constant AUC throughout the different class imbalances shows that all methods are capable of correctly separating both classes. Nonetheless, increasing the amount of samples of the majority class (NC) increases the classification noise at the class boundary, as well as the amount of duplicate samples in feature space belonging to different classes. To illustrate this phenomenon, Fig. 20 shows the ROC curve of the NN classifier trained on Dataset (A) with the original class balance. We can see how the classifier reaches a $\sim 90\%$ true positive rate at about $\sim 10\%$ false positive rate with an AUC of $94.71\%$. Nonetheless, a 10% of the majority class is already twice as large as the whole minority class corpus.

Moving onto early prediction, we drop some of the models from the analysis in order to prevent cluttering the exposition. From the feature-engineering part, we exclude the Random Forest (RF) classifier, provided that it is consistently outperformed by XGB and both are tree-based methods. From the DL side, we exclude the discriminative LSTM with average pooling, provided that it consistently underperforms with respect to its twin model with last pooling.

**Figure 21:**
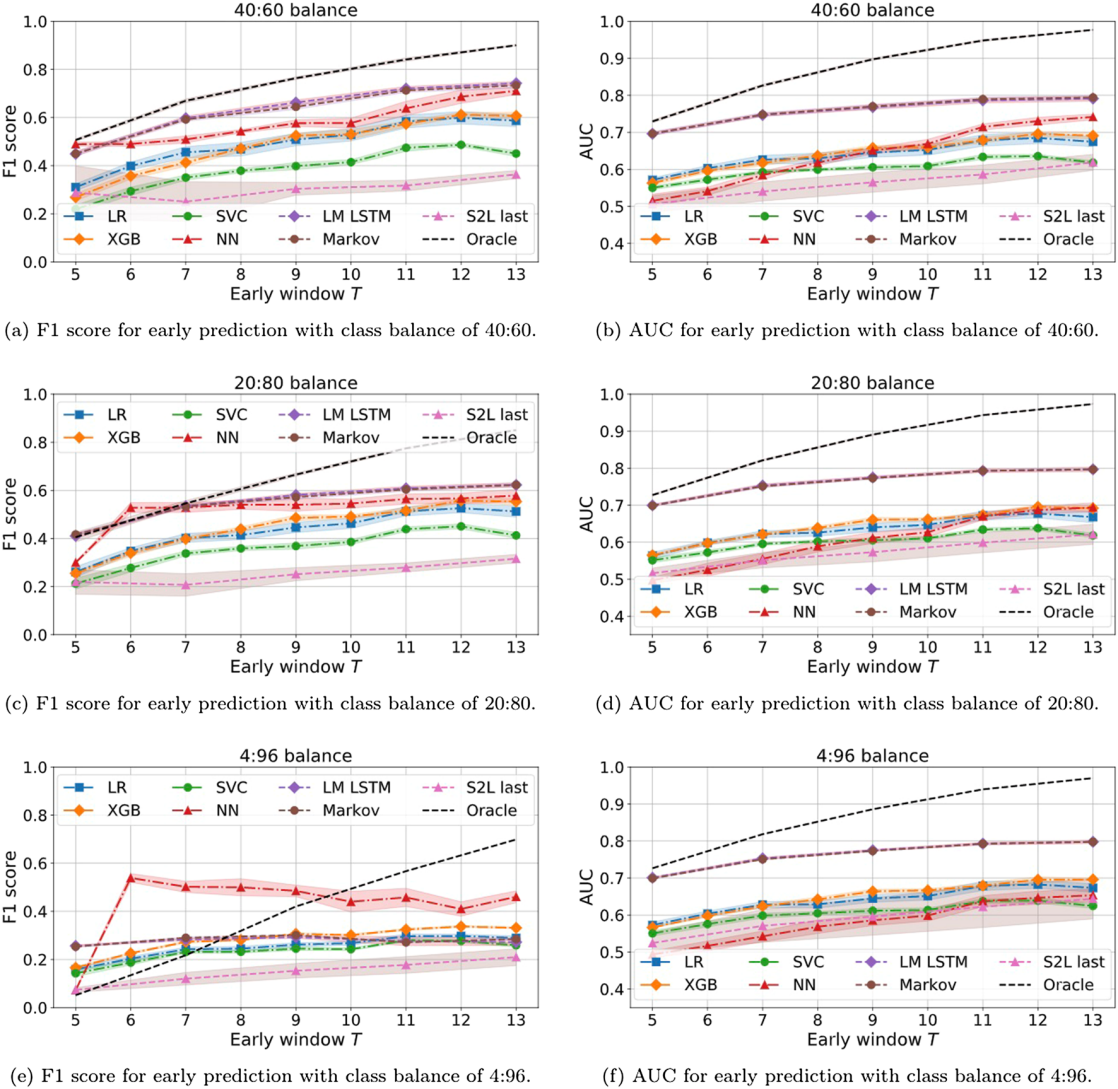
F1 (left) and AUC (right) results on early prediction task in a more realistic scenario. All models are trained to perform full trajectory classification on balanced datasets. Then, they are evaluated on the early prediction task using test sets with different C:NC proportions.

Looking at Fig. 21, the first thing that we notice is that the S2L model with last pooling is, by far, the worst performing method, in contrast to what we saw in full trajectory prediction (Fig. 19). Hence, this is the model that suffers the most when facing a different task from the one for which it is optimized.

The Oracle model is used as upper bound reference. The model knows the true distribution of classes and assigns, to each trajectory, the empirical probability that it belongs to class C. For example, for a trajectory that is repeated five times and ends with a purchase action four times, the model will output 0.8 (4/5). For early prediction, the shorter the early window, the higher the probability of having duplicates, which constitutes one of the major challenges of the task. Notice that, while this model maximizes the accuracy, it does not maximize neither F1 nor AUC, provided that the degree of uncertainty enters into play and class imbalance heavily affects results. We have refrained from plotting the Oracle prediction in Fig. 19 provided that its AUC is close to one and that F1 ranges from 96% to 93% with increasing imbalance, meaning that full trajectories are rather unique.

In terms of F1, the overall performance drops with increasing imbalance. The generative DL-based methods, i.e. the LM LSTM and Markov chain classifier, outperform the feature-engineered models for low imbalance, but the latter ones are more resilient to class proportions. This way, for the original class balance, the DL classifiers end up being matched and outperformed by the handcrafted feature models, being the NN the best classifier for this case. Surprisingly, the peak performance of this model is at an early window of $T=6$, breaking the usual monotonic tendency of the curve.

In terms of AUC, all models show a similar behaviour that is consistent with increasing class imbalance, analogous to the case of full trajectory classification. The generative DL-based models hold the best AUC with barely any differences between their results, while the discriminative model holds the worst results. Surprisingly, the NN classifier fed with hand-crafted features has some of the lowest AUCs but the best F1 scores in the case of high class imbalance.

Overall, the two generative classifiers outperform the rest until high class imbalances, where the NN fed with hand-crafted features takes the lead. These two classifiers seem to suffer less from the discrepancy between the training objective and the test evaluation, as they do not attempt to learn the relationship between sequences of events and classes directly, but, rather, they develop a generative model of how users behave when they aim to purchase and when they do not. However, these models have their own limitations as well, the main one being that a separate model needs to be trained for every different class, with MAP classification on top of it. Therefore, they are appealing when dealing with few classes (as in this case), but do not scale well to tasks which involve too many of them.

Hence, these results highlight a theoretically and practically relevant difference between generative and discriminative models. The former models are slightly less efficient but generalize much better to early prediction, given that they are optimized for a different task than classification, namely, predicting the next event. The latter models, on the contrary, perform best when evaluated on their target task but generalize the worst to classification problems for which they are not specifically optimized. The high performance of discriminative models indicates that there are sequential patterns which are highly discriminative of one class or the other, but the poor generalisation to early prediction when trained on whole trajectories shows that the *most* discriminative patterns are likely to occur towards the end of each sequence. However, having shown good classification performance when including short sequences in training also proves the existence of discriminative patterns early on, which however are likely to differ from and be less predictive than the patterns learned on whole sequences.

Future research should investigate whether the strengths of discriminative and generative models can be combined, for example, using multi-task models which jointly optimize parameters to predict the next event and the sequence class. The feature-based classifiers underperform for full trajectory classification, but they behave better than the DL-based classifiers for early prediction, making them rather appealing for industrial applications. Besides, they are a powerful tool to interpret potentially useful patterns in clickstream sequence that can help to understand the performance of more efficient and opaque models, such as the neural networks. Other directions that could be explored would be training the models to predict at a given early window size and see how they generalize to other inference times, instead of training them with full trajectories.

## Conclusion

In this work, we leveraged a new clickstream dataset from a popular e-commerce website to predict purchase vs. no-purchase user trajectories. Our research has shown that, even by using the most basic information (e.g. event type), it is possible to reliably predict conversion based on simple and lightweight features in a variety of scenarios. As discussed in section "Preliminaries", the goal of *this* work is two-fold: on one hand, provide algorithms that improve the current performance of machine learning systems on the clickstream prediction challenge; on the other, draw a general map of this sequence-classification problem, by providing insights, benchmarks and solid baselines both at the “computational” *and* “implementation” level.

We compared different approaches to sequence modelling, and tackled the clickstream challenge as a full sequence as well as an early prediction scenario: our extensive benchmarks, coupled with model interpretation techniques, allowed us to gain insights about behavioural patterns of e-commerce customers. Finally, we reported deep architectures that improve over previous methods^4^, consistently outperforming them in full trajectory classification, at the cost of increasing training time and more complex engineering infrastructure. Our extensive analysis of generative and discriminative models in the minimal, symbolized setting produced not only an “algorithmic recipe” to solve this particular challenge, but also more general insights on the underlying informational structure, and, possibly, methodological considerations to be ported to other challenges involving symbolized sequences.

Three directions for future work can be outlined. First, improving performance by including more information: by using more sophisticated features and/or by extracting more metadata to build the symbolic sequences. For example, the prediction accuracy is likely to be improved by coupling *k*-grams and other network structural properties associated to the location of the trajectory at each time step. While preliminary experiments with time intervals between events only yielded marginal accuracy improvements, combinations of product metadata and time intervals are worthy of further investigation.

Second, extend to other classification problems: predicting purchase is only one of several user intent prediction problems of relevance for the industry. For instance, other possibilities include cart abandonment prediction, that is, predicting whether a user that has loaded the cart with some products will, ultimately, purchase some of them or leave the session without purchasing anything. This problem is of utmost relevance and can be readily tackled under our framework by focusing on all the trajectories where the Add action, corresponding to adding a product to the cart, appears, at least, once without a Purchase action.

Third, implementing efficient prediction models, such as the ones described in this paper, for online testing. By doing so, the algorithm could proactively detect customers which have a high probability of leaving the session without purchasing and, then, for instance, implement targeted marketing strategies to boost their conversion likelihood.

As a final note, it is important to mention a recent debate in the machine learning community, in which more and more researchers pointed out unfairness^47^ and inefficiency in the current research agenda dominated by large DL models: on one hand, the carbon footprint of specialized hardware (i.e. GPUs) is high; on the other, training and testing infrastructure make developing research papers prohibitively costly for scientists outside a few tech companies. While in this work we remained neutral on these arguments, we do believe that they strengthen our earlier observation on the difficulty for the community to generalize the findings of many research papers, even if datasets were available and were indeed representative of the industry at large (which, as we discussed, was not the case with^4^). Sometimes, even just model complexity and scale will make the proposed solutions not feasible for many companies/institutions. In this spirit, we find it important to remark that visibility graphs provide a simple method for sequence predictions at minimal deployment cost, and that our thorough benchmarks across architectures and scenarios provide a solid quantitative starting point to discuss the *marginal* value of deep learning architectures for clickstream prediction in a variety of business contexts.

## Data Availability

*Dataset*: As part of Coveo’s ongoing mission to help the retail space leverage the latest A.I. techniques and to promote multidisciplinary research in data science across industries, Coveo is working with its legal advisors to release a substantial, anonymized subset of the dataset used in this study under a research-friendly license. For preliminary enquiry, please reach out to the authors.
